# Transgenic phytochrome D cooperates with phytochrome B to promote photomorphogenesis at low temperatures

**DOI:** 10.1007/s00299-026-04025-y

**Published:** 2026-10-23

**Authors:** Lilla Sípos, Gábor Grézal, Csaba Péter, Ferenc Nagy, Dániel Silhavy, András Viczián

**Affiliations:** 1 Laboratory of Photo and Chronobiology, Institute of Plant Biology, Biological Research Centre, Hungarian Research Network (HUN-REN), Szeged, 6726 Hungary; 2 Faculty of Sciences and Informatics, Doctoral School of Biology, University of Szeged, Szeged, 6726 Hungary; 3 Metabolic Systems Biology Laboratory, HCEMM–BRC, and Genomic Epidemiology Research Group, HUN-REN-BRC-ELTE-SZTE-NNGyK, Szeged, 6726 Hungary; 4 Synthetic and Systems Biology Unit, Biological Research Centre, Institute of Biochemistry, Hungarian Research Network (HUN-REN), Szeged, 6726 Hungary

**Keywords:** Arabidopsis, Light signaling, Photomorphogenesis, Phytochrome B, Phytochrome D

## Abstract

**Key message:**

**The function of the phytochrome B photoreceptor is enhanced by its homologous counterpart, phytochrome D, at low temperatures, thereby increasing phenotypic plasticity in response to fluctuating environmental conditions.**

**Abstract:**

Light is a dynamically changing environmental factor that influences nearly every aspect of plant growth and development. Photoreceptors detect light, with phytochromes (PHY) specifically monitoring red and far-red wavelengths. *Arabidopsis thaliana* has five phytochromes, named phyA through phyE. phyA and phyB are extensively studied key mediators of most phytochrome responses; the precise functions of the other phytochromes remain poorly understood. To investigate phyD signaling, we overexpressed phyD-YFP alone and together with phyB-CFP in a genetic background lacking other phytochromes. Photomorphogenic responses, protein accumulation, and transcriptome changes were compared at low and high temperatures. We found that phyD-YFP alone cannot facilitate photomorphogenesis, but it significantly promotes red light signaling of both phyB-CFP and endogenous phyB in a temperature-dependent manner, being effective at low but not at high temperatures. In parallel, co-expressing phyD-YFP and phyB-CFP triggered substantially more robust light-induced transcriptome changes at low temperatures than expressing either transgene alone. Because phyD-YFP is more light-stable than phyB, and both PHYB-CFP and endogenous PHYB accumulate to higher levels alongside PHYD-YFP in light, phyD likely stimulates phyB signaling via PHYB protein stabilization. To explain how PHYD-YFP enhances PHYB levels at both temperatures yet stimulates signaling exclusively at low temperatures, we propose that phyD and phyB form light-stable heterodimers at both temperatures and that these heterodimers are functional only at low temperatures. Our findings demonstrate that phyB and phyD functionally cooperate to mediate temperature-dependent light signaling responses, providing plants with developmental flexibility under changing environmental conditions.

**Supplementary Information:**

The online version contains supplementary material available at https://doi.org/10.1007/s00299-026-04025-y.

## Introduction

Plants, as sessile organisms, rely entirely on light for energy through photosynthesis, meaning their growth must be optimized according to the light environment around them. In the absence of light, seedlings undergo skotomorphogenesis, characterized by elongated hypocotyls and small, closed cotyledons. However, when exposed to light, photomorphogenesis takes place, resulting in short hypocotyls and expanded, green cotyledons. To detect light, plants possess specialized photoreceptors that respond to a wide range of wavelengths (Briggs and Christie 2002; Demarsy and Fankhauser 2009; Rizzini et al. 2011; Wang and Lin 2020). Among these photoreceptors, the phytochromes primarily detect red (R, *ʎ*_max_~660 nm) and far-red (FR, *ʎ*_max_~730 nm) light. In *Arabidopsis thaliana* five phytochromes (phyA-phyE) were identified (Quail 2002; Bae and Choi 2008). These proteins function as dimers of two 125 kDa monomers each of them binding a linear tetrapyrrol chromophore responsible for light sensitivity. Phytochromes are synthesized in an inactive form (Pr), and upon absorbing red light photons, they convert to the biologically active Pfr form (Rockwell et al. 2006), while far-red light quickly reverts Pfr to the inactive Pr. The thermodynamically unstable Pfr can also revert back to Pr in a light-independent manner in a process known as thermal reversion, which attenuates light signaling (Klose et al. 2020; Viczián et al. 2020). High temperature accelerates thermal reversion, and thus moderate plant developments via the stability of the active phytochrome conformer, allowing phytochromes to act as thermosensors (Jung et al. 2016; Legris et al. 2016). Light-activated phytochromes translocate to the nucleus and form protein complexes, so-called photobodies (Kim et al. 2023; Du et al. 2026). These complexes target specific genomic regions to regulate transcription, linking light perception with gene expression (Chen et al. 2014; Jung et al. 2016; Péter et al. 2024).

The available amount of phytochromes for signaling is also regulated by their protein stability. phyA is the least stable phytochrome in light (Type I phytochrome), thus, it predominantly mediates responses in dim light and continuous far-red light. The Type II (phyB-E) phytochromes are responsible for classical red/far-red light reversible responses. These phytochromes are more stable in light than phyA. However, because the Pfr form of phyB (the dominant Type II phytochrome) degrades faster than its Pr form, phyB levels are higher in dark-grown than in light-grown plants (Nagy and Schäfer 2002; Ni et al. 2014).

Dimer formation is essential for the phytochrome function. phyA appears exclusively as homodimers, but Type II phytochromes form homodimers as well as a variety of heterodimers. phyB forms heterodimers with phyC, phyD, and phyE, and also phyD/phyE heterodimers were observed. Although heterodimers have been described decades ago, very little is known about the biological function of different phytochrome heterodimers and the regulation of heterodimerization (Clack et al. 2009; Ádám et al. 2013; Liu and Sharrock 2013; Viczián et al. 2020).

Whereas phytochrome signaling is dominated by the very abundant phyA and phyB, the less abundant phyC-E have subtler but still significant roles in light regulation mediating diverse responses (Aukerman et al. 1997; Devlin et al. 1998, 1999; Franklin et al. 2003; Halliday and Whitelam 2003; Monte et al. 2003; Strasser et al. 2010; Hu et al. 2013; Sánchez-Lamas et al. 2016; Li et al. 2026). Because phyC-E are expressed at low levels, their overexpression in a protein tagged form is a key approach to examine their function (Kircher et al. 2002; Sharrock et al. 2003; Fernández et al. 2005; Ádám et al. 2013; Sánchez-Lamas et al. 2016; Viczián et al. 2020; Péter et al. 2024). Moreover, as phytochromes are partially redundant, higher order phytochrome null mutants are used to examine the roles of individual phytochromes and separate their redundant functions (Strasser et al. 2010; Sánchez-Lamas et al. 2016; Hu and Lagarias 2025).

PhyD is the closest homologue of phyB, resulting from a gene duplication that occurred after the divergence of Brassicaceae family (Mathews 2005; Mathews and McBreen 2008). Despite their high sequence homology, phyB and phyD exhibit distinct roles; phyB is the major red light responsive photoreceptor that accumulates to high levels, whereas phyD levels are lower (Sharrock and Clack 2002), and it plays only minor regulatory role in various developmental processes, such as germination, seedling de-etiolation, and flowering (Sharrock et al. 2003; Strasser et al. 2010; Ádám et al. 2013; Sánchez-Lamas et al. 2016; Péter et al. 2024; Hu and Lagarias 2025). Interestingly, under light exposure, phyD is much more stable than phyB (Péter et al. 2024). Studies using transgenic Arabidopsis lines overexpressing phyD-YFP demonstrated that phyD alone mediates very limited light responses. However, particularly at low temperatures, phyD cooperates efficiently with phyC and regulates a characteristic set of genes involved in seedling development and floral induction (Viczián et al. 2020; Péter et al. 2024). This finding that PHYD-YFP synergistically interacts with phyC in a temperature-dependent manner inspired us to analyze whether it can also collaborate with other phytochromes.

To investigate whether phyD can also cooperate with phyB, we overexpressed tagged phyD together with tagged phyB in a quintuple mutant background or with endogenous phyB in a mutant Arabidopsis background lacking all other endogenous phytochromes. Photomorphogenesis and transcriptome assays have shown that phyB and phyD photoreceptors synergistically cooperate in light signaling, and that phyD contribute to the signaling mainly at low temperatures. Our results also indicate that phyD-YFP stabilizes phyB in irradiated seedlings. Collectively, phyD maintains active phyB amounts at low temperature thereby enhancing phytochrome-mediated plant responses to specific environmental conditions.

## Materials and methods

### Plant materials, growth conditions and seedling phenotyping

Phytochrome mutants in *the Arabidopsis thaliana* (L) Ler ecotype were obtained by genetic crossings. The names of higher order phytochrome mutants are abbreviated as follows: abcde: (*phyA-201phyB-1phyC-1phyD-1phyE−1*), aBcde: (*phyA-201phyC-1phyD-1phyE−1*) (Hu et al. 2013; Péter et al. 2024). Generation of the transgenic lines *35S:PHYD-YFP*/abcde, *35S:PHYD-YFP*/abCde and *PHYB:PHYB-CFP*/abcde was described earlier (Péter et al. 2024). These lines were crossed to obtain *35S:PHYD-YFP* + *PHYB:PHYB-CFP*/abcde (abbreviated as:PHYD-YFP + PHYB-CFP throughout the study) and *35S:PHYD-YFP/aBcde.*

Seeds were surface sterilized, placed on half strength MS agar plates, and kept at 4 °C for 4 days. After 6 h of white light irradiation (22 °C, 100 μmol m^−2^ s^−1^, Lumilux XT T8 L 36 W/865 fluorescent tubes; Osram) seeds were grown in the dark or under red light (660 nm, Snap-Lite; Quantum Devices) at either 17, 22 or 27 °C as described at each experiment.

For the determination of hypocotyl length, seeds were either grown as written above or were sown on 4 layers of wet filter paper and after 4 days of stratification at 4 °C in the dark, germination was induced using 6 h of white light at 22 °C. The seedlings were grown in the dark or under constant red light irradiation at the indicated temperature. Measurement of hypocotyl length and data processing have been described previously (Ádám et al. 2013; Dobos et al. 2019).

### Protein isolation, co-immunoprecipitation and immunoblot assays

Preparation of plant protein extracts and immunoblot analysis were done has been described in detail (Vanhaelewyn et al. 2019; Péter et al. 2024). The following primary antibodies were used: anti-GFP (Clontech), anti-ACTIN (Sigma-Aldrich), anti-HA (Roche), anti-PHYB (Agrisera) together with the corresponding Polyclonal Swine Anti-Rabbit Immunoglobulins/HRP (Dako, Glostrup, Denmark) and Goat Anti-Mouse IgG Peroxidase Conjugated antibody (Invitrogen) secondary antibodies. Co-immunoprecipitation was performed using µMACS Anti-GFP Isolation Kit according to the instructions of the manufacturer (Miltenyi Biotec, Bergisch Gladbach Germany). The protein binding was done on ice for 1 h.

### Transcriptomic analysis

Seedling propagation and data processing was performed as described earlier (Péter et al. 2024). Four-day-old seedlings were grown in the dark on half-strength MS plates at 17 °C or 27 °C, as previously described and were seedlings exposed to 1 h or 24 h of R light (50 µmol m⁻^2^ s⁻^1^). Total RNA was extracted from 0.1 g plant material using RNeasy Miniprep Kit (Qiagen) following the manufacturer’s protocol. RNA sequencing was performed by Novogene (Cambridge, United Kingdom).

Data processing was done as described earlier (Péter et al. 2024). Briefly, high-quality reads were obtained using FastQC 0.11.9 (https://www.bioinformatics.babraham.ac.uk/projects/fastqc/) and TrimmomaticPE 0.39 (Bolger et al. 2014) with the following parameters: TruSeq3-PE−2 adapters were cut from the reads with 2:30:10 values. The bases were cut from the end of a read if below 5 quality score. The reads were trimmed from the 5’ end if the average quality dropped below 20 in a 5-base-pair-long sliding window Reads shorter than 50 base pairs or with an overall quality score below 20 were discarded. Read alignments against the *Arabidopsis thaliana* reference genome (https://www.arabidopsis.org, TAIR10 chromosome files) was performed using STAR 2.7.8a (Dobin et al. 2013), followed by coordinate sorting with Samtools 1.11 (Danecek et al. 2021). Gene-level count data across the Arabidopsis genome annotation (https://www.arabidopsis.org, TAIR10 GFF3) were derived using htseq-count 0.13.5 (Anders et al. 2015). Subsequently, tRNA, rRNA, mitochondrial and chloroplasts genes were discarded. In addition, low-abundance genes showing fewer than 5 counts across over 75% of the samples were omitted from downstream analysis. To evaluate light-induced gene expression changes, log-transformed, quantile-normalized values were evaluated, with dark-kept plants serving as baseline controls across all genotypes, light conditions, and temperature treatments Finally, log2 fold changes and FDR-adjusted *p* values (*q* values) were calculated using the edgeR 3.38.4 package (Robinson et al. 2010; Huber et al. 2015).

### Arabidopsis gene accession numbers

PHYB, AT2G18790; PHYD, AT4G16250.

## Results

### phyD-YFP mediate photomorphogenesis together with phyB-CFP and endogenous phyB at low but not at high temperature

**Fig. 1:**
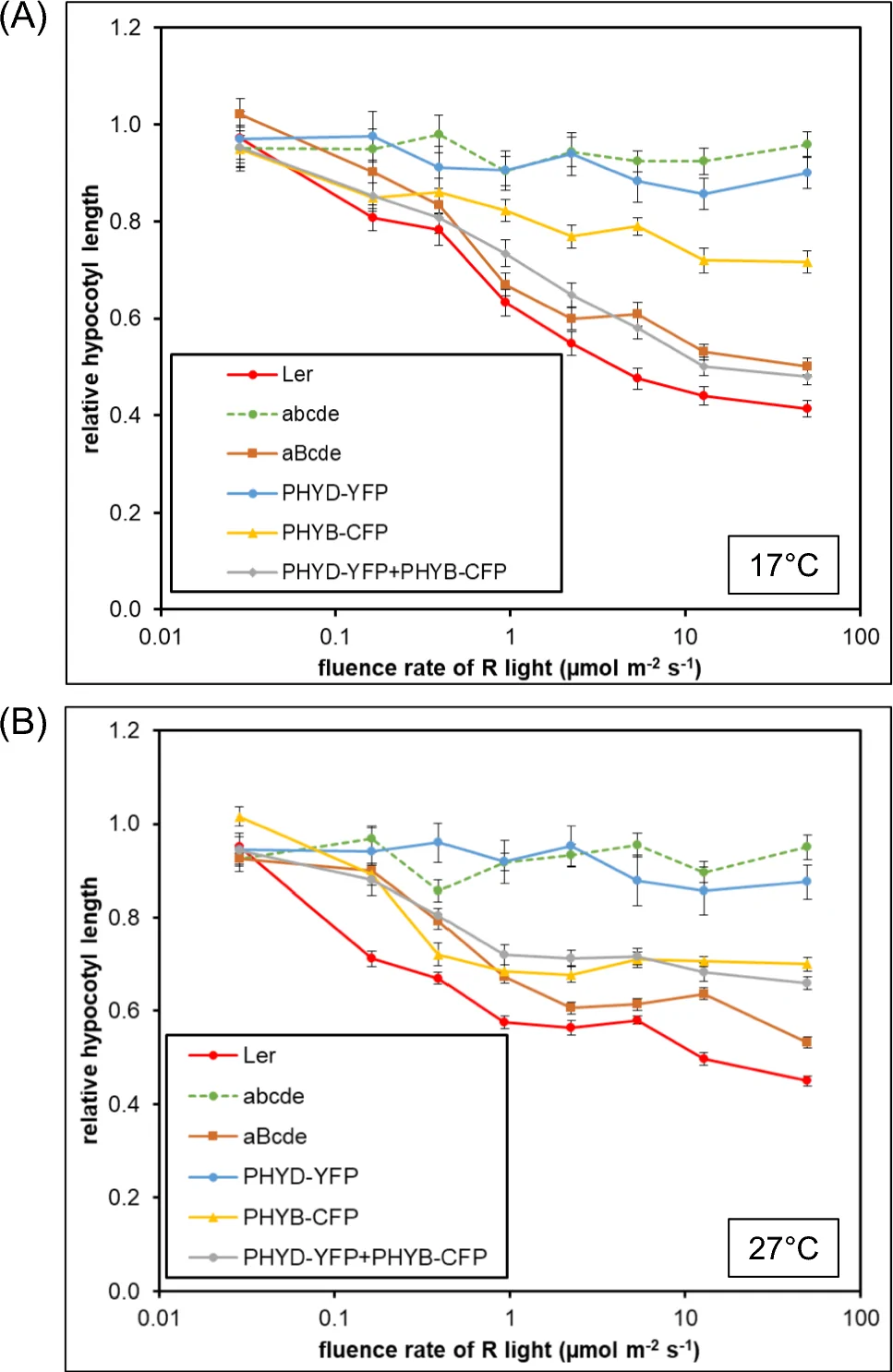
phyD-YFP together with phyB-CFP effectively modulates seedling photomorphogenesis at low temperatures. Seedlings were grown for 4 days under different fluences of constant R light irradiation at either 17 °C **A** or at 27 °C **B**. Hypocotyl lengths were measured and the values were calculated and depicted relative to the corresponding dark controls. *n* ≥ 30, error bars indicate standard errors

To examine the physiological role of phyD in photomorphogenesis and overcome the problem of low levels and poor detectability of the endogenous proteins, we generated transgenic Arabidopsis plants. We overexpressed YFP-tagged phyD at high protein levels under the control of the strong constitutive *35S* promoter in a genetic background lacking functional phytochromes (*35S:PHYD-YFP*/abcde, referred to as PHYD-YFP). We also generated a transgenic line expressing CFP-HA-tagged PHYB under the control of its native *PHYB* promoter in the same phytochrome-less genetic background (*PHYB:PHYB-CFP*/abcde, referred to as PHYB-CFP) (Supplementary Fig S1). We then crossed these lines to obtain *35S:PHYD-YFP* + *PHYB:PHYB-CFP*/abcde double transgenic plants (referred to as PHYD-YFP + PHYB-CFP) that express both PHYD-YFP and PHYB-CFP but no endogenous phytochromes. To assess the function of these transgenes, we grew the seedlings under constant red light (R) irradiation at low (17 °C) or high (27 °C) ambient temperature, and measured their R signaling using inhibition of hypocotyl elongation photomorphogenesis assay. As controls, wild-type plants and mutants, which do not express phytochromes (abcde) or produce only endogenous phyB (aBcde) were used. Figure 1 shows that phyD-YFP alone was unable to inhibit hypocotyl elongation at either temperature, as previously described by (Péter et al. 2024) and phyB-CFP could mediate photomorphogenesis at both temperatures, although at strong R, to a lesser extent than the endogenous phyB alone (aBcde plant), which is in agreement with our previous findings that, on norflurazone-containing media R signaling was less efficient in PHYB-CFP/abcde than in aBcde plants (Péter et al. 2024). Figure 1 also shows that phyD-YFP and phyB-CFP acted synergistically in a temperature-dependent manner, with the inhibition of hypocotyl elongation being more effective in PHYD-YFP + PHYB-CFP than in PHYB-CFP plants at 17 °C, but not at 27 °C. Strikingly, at 17 °C the PHYD-YFP + PHYB-CFP inhibited hypocotyl elongation to the same extent as the endogenous phyB, and almost as efficiently as the wild-type seedlings (Fig. 1). To test, whether phyD-YFP can also cooperate with endogenous phyB, we crossed *35S:PHYD-YFP* transgene into the aBcde line. These PHYD-YFP/aBcde seedlings also exhibited enhanced photomorphogenesis compared with the aBcde control at low but not at high temperatures under strong R irradiation (Supplementary Fig S2A and B).

Thus, overexpressed phyD-YFP enhances both transgenic and endogenous phyB-mediated photomorphogenesis at low temperatures indicating that phyD-YFP and phyB phytochromes cooperate in a temperature-dependent manner.

### phyD-YFP increases PHYB-CFP and endogenous PHYB protein levels

Photoreceptor protein abundance affects the efficiency of light signaling. Thus, we measured protein expression and compared these protein levels to the photomorphogenesis phenotypes. First, we compared the expression of the endogenous PHYB and the transgenic PHYB-CFP. We found that endogenous phyB accumulated to higher levels in the aBcde plant than PHYB-CFP in the PHYB-CFP/abcde or in the PHYD-YFP + PHYB-CFP/abcde plants (Supplementary Fig S3), which explains why R signaling was more efficient in the aBcde seedlings (Fig. 1). We also noticed that PHYB-CFP amounts were higher in the double than in the single transgenic line.

**Fig. 2:**
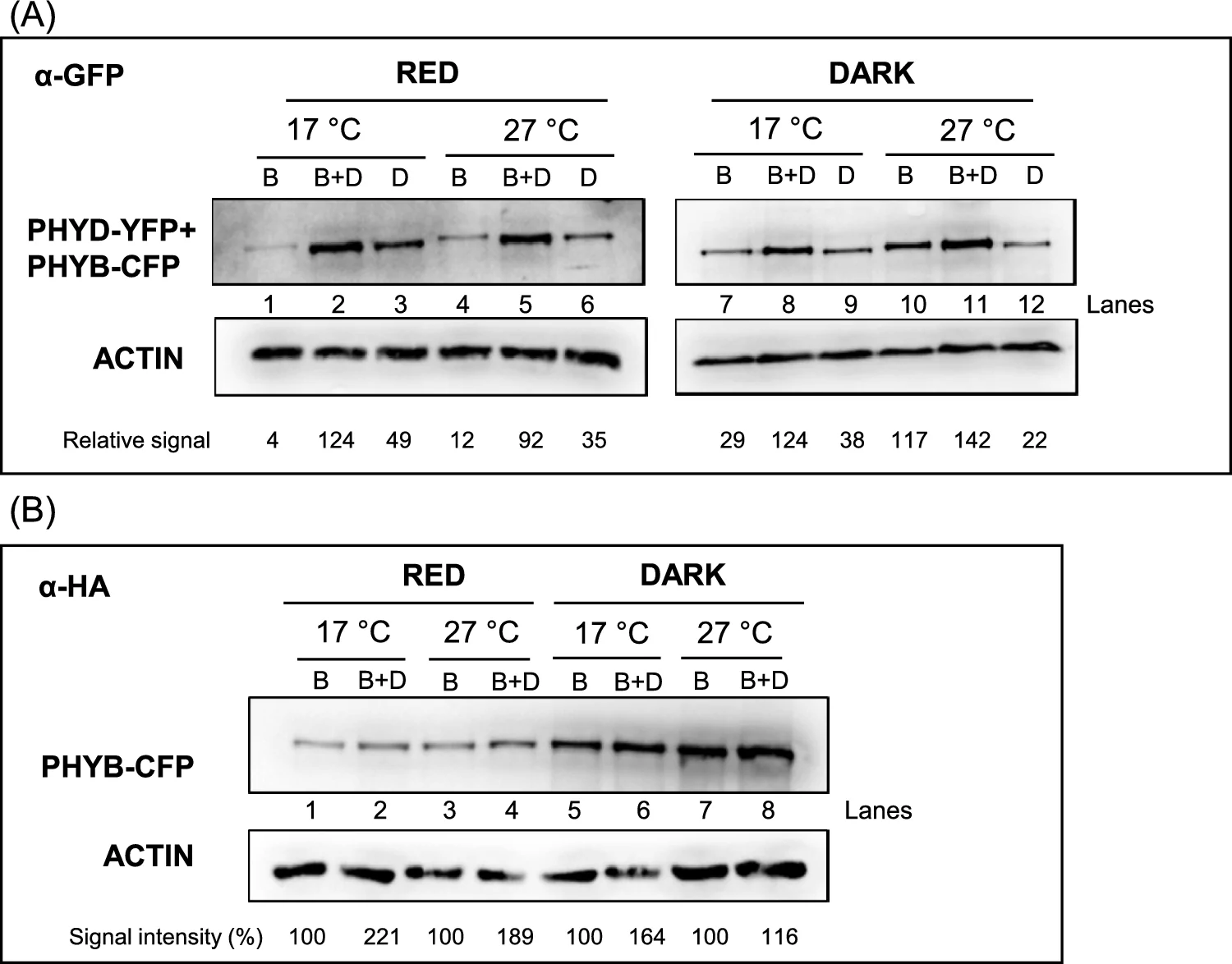
Accumulation of PHYD-YFP and PHYB-CFP proteins show similar pattern at low and high temperatures. Seedlings expressing PHYD-YFP (D), PHYB-CFP (B), or both PHYD-YFP and PHYB-CFP (B + D) in phytochrome-deficient background (abcde) were grown under constant red light (50 µmol m^−2^ s^−1^) or in darkness at 17 or 27 °C for four days. Total protein extracts were tested by immunoblotting using anti-GFP antibody, that detects both PHYB-CFP and PHYD-YFP chimeric proteins (**A**) or the anti-HA antibody that detects PHYB-CFP (**B**). anti-ACTIN antibody was used as a loading control. Numbers below the blots indicate band intensities normalized to the corresponding ACTIN signal (**A**), or normalized to ACTIN with the dark sample for each genotype and temperature condition set to 100% (**B**)

Next we compared PHYB-CFP and PHYD-YFP protein amounts in etiolated and red light-grown transgenic seedlings at low and high temperatures (Fig. 2 and Supplementary Fig S4). The used anti-GFP antibody recognizes both PHYD-YFP and PHYB-CFP, whereas anti-HA detects only PHYB-CFP due to the presence of a HA tag at the C-terminus of the CFP (Supplementary Fig S1).

**Fig. 3:**
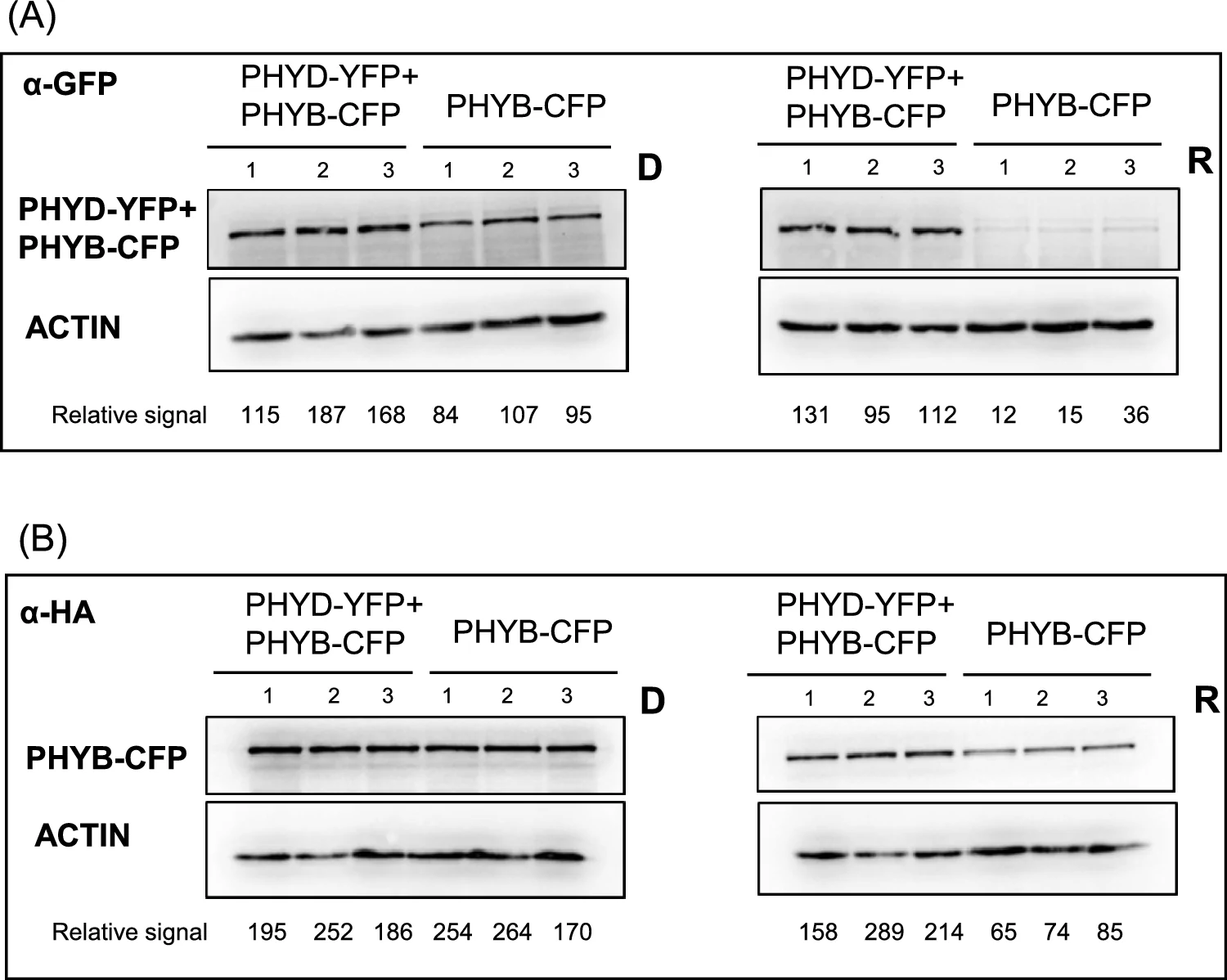
PHYD-YFP dominates the phytochrome pool and stabilize phyB-CFP when co-expressed with PHYB-CFP in red light. Seedlings were grown at 17 °C for 96 h in darkness (D) or in 50 µmol m^−2^ s^−1^ red light (R). Total protein extracts were probed in western blot hybridization using anti-GFP (panel **A** which detects both PHYD-YFP and PHYC-CFP) or using an anti-HA antibody (panel **B** which detects only PHYB-CFP). Hybridization with anti-ACTIN was performed as a loading control. Numbers above the blots indicate biological replicates. Numbers below the blots indicate band intensities normalized to the corresponding ACTIN signal

First we analyzed the protein levels at 17 °C, where photomorphogenesis is more efficient in the PHYD-YFP + PHYB-CFP than in the PHYB-CFP plants. In line with previous reports (Ni et al. 2014), we found that, the phytochrome level decreased dramatically under prolonged R irradiation in the PHYB-CFP line (Fig. 2B compare lanes 1 with 5, Supplementary Fig S4A and B, compare lanes 5 with 8). One hour of R induced only a small decrease, whereas after 24 h, PHYB-CFP levels were similar to those observed in seedlings grown under constant R for 4 days (Supplementary Fig S4, compare lanes 5 with 6–8). Confirming our previous findings (Péter et al. 2024), we observed that PHYD-YFP accumulates to higher levels than PHYB-CFP under R irradiation (Fig. 2A compare lanes 1 with 3 and 7 with 9). We have previously also shown that PHYD-YFP protein level decreased only slightly during 4 days of R (Péter et al. 2024). The PHYD-YFP + PHYB-CFP plants contain more phytochromes than those seedlings that express only PHYB-CFP. This difference is drastically higher in the red light-grown PHYD-YFP + PHYB-CFP seedlings than in the etiolated plants (Fig. 3 and Supplementary Fig S4A compare lanes 1 with 5 and 4 with 8). Thus, PHYD-YFP constitutes the majority of the phytochrome pool in the PHYD-YFP + PHYB-CFP plants under this condition (prolonged R at low temperatures), where synergistic phytochrome interaction was found. Relevantly, the presence of PHYD-YFP also modifies the accumulation of PHYB-CFP. We have found that the amount of PHYB-CFP is higher under R, but not in darkness, when PHYD-YFP is present (Fig. 3 and Supplementary Fig S4B compare lanes 4 with 8 and 1 with 5, and Fig. 2B). This interesting observation suggests that PHYD-YFP stabilize PHYB-CFP in red light, a condition under which phyB degradation is enhanced. To extend these findings, we measured the amounts of endogenous phyB under the same conditions (R irradiation, low temperature) in the PHYD-YFP/aBcde line and found that phyD-YFP can also stabilize the endogenous phyB (Supplementary Fig S2C). Previously we have shown that under constant R irradiation, PHYD-YFP protein abundance is dramatically increased in the presence of endogenous phyC (Péter et al. 2024). Surprisingly, endogenous phyB did not increase the PHYD-YFP amounts under the same conditions (Supplementary Fig S5).

Collectively, our results indicate that at low temperature PHYD-YFP increases phyB protein abundance in a light-dependent manner, which might explain how it enhances phyB-mediated light signaling.

We also compared the accumulation of phytochromes in seedlings grown at high temperatures. As we reported earlier (Péter et al. 2024), PHYD-YFP accumulates to lower levels at high than at low temperatures in the PHYD-YFP plants (Fig. 2A compare lanes 3 with 6 and 9 with 12). By contrast, PHYB-CFP levels were higher at 27 °C than 17 °C in both dark and red light grown plants (Fig. 2A compare lanes 1 with 4 and 7 with 10). Then we analyzed the protein levels in the double transgenic plants and found that PHYD-YFP comparably enhanced PHYB-CFP levels in red light-grown plants at both low and high temperatures (Fig. 2B, compare lanes 1 with 2 and 3 with 4). This effect was light-dependent, in etiolated seedlings PHYD-YFP does not alter the PHYB-CFP levels at either temperature (Fig. 2B, compare lanes 5 with 6 and 7 with 8).

Taken together, these results show that in continuous red light-grown double transgenic plants, PHYD-YFP comparably promotes the accumulation of PHYB-CFP protein at low and high temperatures, although PHYD-YFP accumulates to higher levels at low temperature and stimulates photomorphogenic response only at 17 °C.

### phyD-YFP and phyB-CFP cooperate in light-dependent transcriptome reprogramming only at low temperature

Comparative RNA-seq assays were conducted to reveal (i) why PHYB-CFP plants mediate signaling less efficiently than aBcde plants under strong R irradiation, and to understand (ii) how phyD-YFP promotes R signaling at low but not at high temperatures in the PHYD-YFP + PHYB-CFP plants.

We performed RNA-seq on samples isolated from PHYB-CFP and PHYD-YFP + PHYB-CFP plants, which were grown for 4 days in the dark at 17 or 27 °C (control samples). Additionally, we analyzed 4-day-old dark-grown plants that were treated with strong R light for 1 or 24 h. This high-intensity irradiation was chosen because it yielded the most distinct phenotypic differences between the genotypes. Then we compared these data to the results of RNA-seq of identically grown and treated aBcde and PHYD-YFP plants (Péter et al. 2024).

**Fig. 4:**
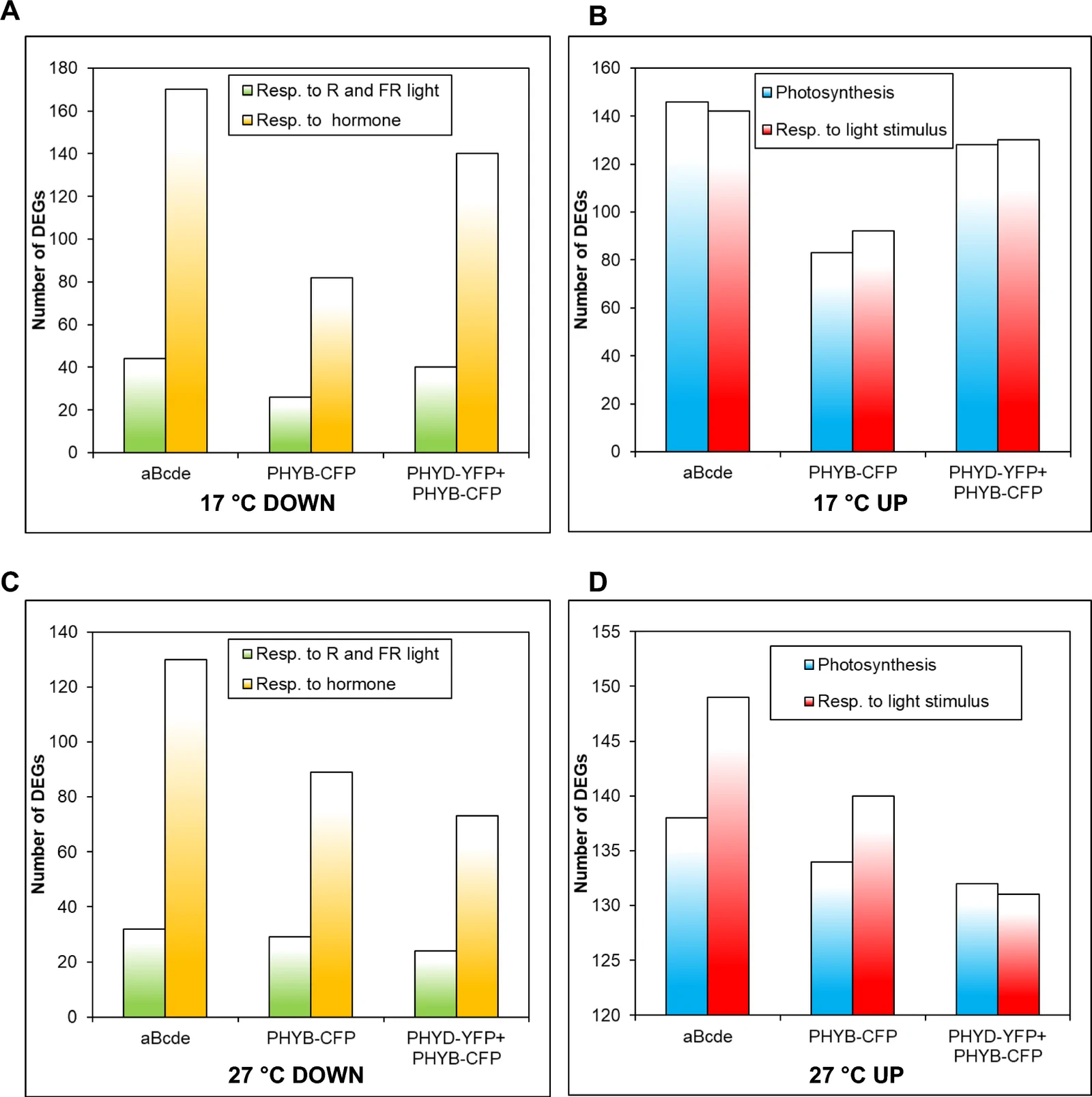
Number of significantly altered genes of the most relevant GO categories in the different red light-treated plants. Number of down- and up-regulated DEGs of the most relevant GO categories at 17 °C (**A**, **B**) and 27 °C (**C**, **D**). GO analysis was conducted with the down- and up-regulated DEGs of the aBcde, PHYB-CFP and PHYD-YFP + PHYB-CFP plants. We selected photomorphogenesis–related GO categories, which were overrepresented in the down- and up-regulated DEGs in all three plant lines. Note that more DEGs were identified in aBcde than in PHYB-CFP plants at both temperatures and that, the number of DEGs of PHYD-YFP + PHYB-CFP plants are at low temperature similar to aBcde plants

Principal component analysis (PCA) showed that the repeats are well grouped, the low and high temperature grown samples are clearly separated and the transcriptome changes are moderate after 1 h but strong after 24 h of treatment (Supplementary Fig S6). After 1 h R treatment, the number of differentially expressed genes (DEG, defined as genes that show significant and at least two-fold expression differences relative to the dark control) varied strongly (*e.g.,* in aBcde plants, at 17 and 27 °C, 1450 and 241 DEGs were found, respectively), and the gene expression changes correlated poorly (Supplementary Fig S7A and B). In contrast, after 24 h R treatment, large numbers of DEGs were identified in all plants, and the transcriptome changes showed strong correlations (Supplementary Tables S1–S9, Supplementary Fig S7A and C). Thus we analyzed further the transcriptome changes of the 24 h R treated plants. First, we compared the transcriptome changes of aBcde and PHYB-CFP plants. As both lines can mediate photomorphogenesis at low as well as at high temperature (although photomorphogenesis was more efficient in aBcde) (Fig. 1), we expected that similar transcriptional reprogramming occur in both plants at both temperatures. Indeed, although many more DEGs were found in the aBcde plants, the DEGs of the two genotypes strongly overlapped (Supplementary Fig S8), and their transcriptome changes robustly correlated at both temperatures (Pearson correlations at 17 and 27 °C, *r* = 0.71 and 0.86, Supplementary Fig S7B and C). Moreover, GO analyzes revealed that similar light response-related GO categories were overrepresented in the aBcde and PHYB-CFP DEGs (Fig. 4 and Supplementary Table S9). The genes of the Response to hormone and Response to red and far-red light GO categories were over-represented in the down-regulated DEGs (Down DEG) in both plants, while genes belonging to the photosynthesis and response to light stimulus GO categories were overrepresented in the up-regulated DEGs (Up DEG). However, consistently with the finding that aBcde mediates R signaling more efficiently at both temperatures, in the aBcde plants many more DEGs were identified (at 17 °C: 3561 vs. 1529 and at 27 °C: 2715 vs. 2191) (Supplementary Fig S7A). Moreover, in the most relevant common GO categories (Fig. 4 and Supplementary Table S9), more genes were significantly modified in the aBcde than in the PHYB-CFP plants (e.g., at 17 °C, 40 and 25 genes of the Response to red and far red light GO category were Down DEGs in the aBcde and the PHYB-CFP plants). We also observed that the transcriptome differences of the aBcde and the PHYB-CFP plants were higher at 17 than 27 °C (Supplementary Figures S6–S9). Taken together, although R leads to similar transcriptional reprogramming in both plants at both temperatures, the transcriptome changes are stronger at both temperatures in the aBcde plants, which manifest in more efficient photomorphogenic responses (Fig. 1).

Next, we analyzed why PHYD-YFP promotes photomorphogenesis in the PHYD-YFP + PHYB-CFP plants only at low temperature. Consistently, we found that R induced transcriptional reprogramming was more efficient in the PHYD-YFP + PHYB-CFP plants at low temperature; many more DEGs were identified in the PHYD-YFP + PHYB-CFP than in the PHYB-CFP plants at 17 °C, while the DEG numbers were comparable at 27 °C (2807 vs. 1529 and 2073 vs. 2191, Supplementary Fig S7A). Moreover, Pearson correlation data and heat-map show that after 24 h R treatment the gene expression changes of the PHYD-YFP + PHYB-CFP plants were more similar to aBcde than to PHYB-CFP plants at 17 °C (Supplementary Figs S7C and S9). In contrast, the transcriptome changes of the PHYD-YFP + PHYB-CFP plants were more similar to the PHYB-CFP than to the aBcde plants at 27 °C (Fig. 4 and Supplementary Figs S6–S9). Relevantly, the GO analyses revealed that at 17 °C but not at 27 °C, higher number of light response-related genes was significantly modified in the PHYD-YFP + PHYB-CFP than in the PHYB-CFP plants (Fig. 4). These results that the phyD-YFP only promotes the R-induced transcriptional reprogramming in the PHYD-YFP + PHYB-CFP plants at low temperature explains the efficient photomorphogenic responses of the PHYD-YFP + PHYB-CFP plants at 17 °C.

### phyD-YFP supports phyB-CFP and endogenous phyB function under R light pulses

**Fig. 5:**
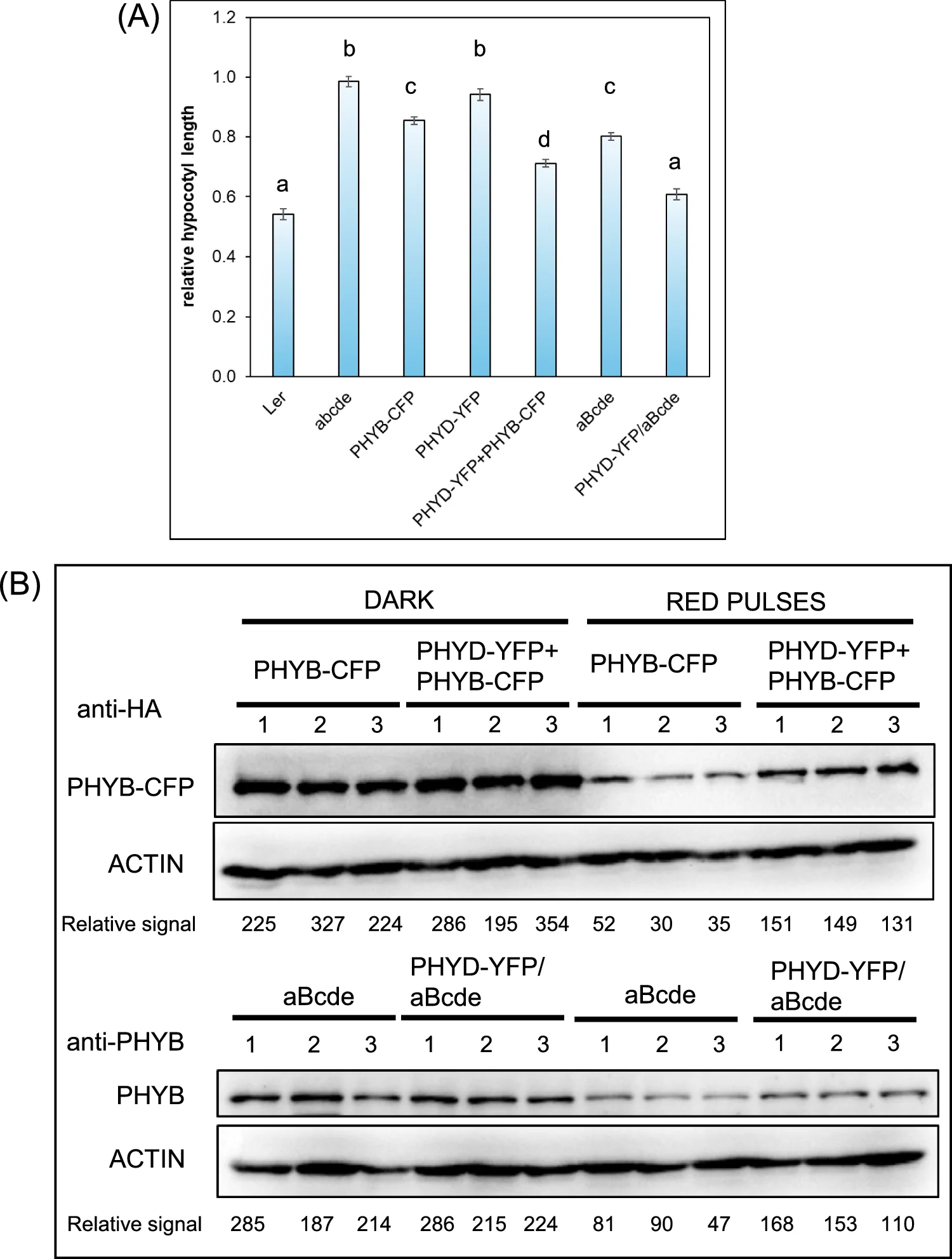
phyD-YFP mediates signaling together with phyB under light pulses. Arabidopsis seedlings were grown at 17 °C in the dark or in the dark, interrupted by 1 min red light (50 µmol m^−2^ s^−1^) pulses in every 3 h for 4 days. The genotypes are: wild-type (Ler); *phyAphyBphyCphyDphyE (*abcde); *35S:PHYD-YFP*/abcde (PHYD-YFP); *PHYB:PHYB-CFP*/abcde (PHYB-CFP); *35S:PHYD-YFP* + *PHYB:PHYB-CFP*/abcde (PHYD-YFP + PHYB-CFP); *phyAphyCphyDphyE* (aBcde); *35S:PHYD-YFP*/aBcde. **A** Hypocotyl lengths were measured, and their values to the corresponding dark controls are depicted. *n* ≥ 35, error bars indicate standard errors. Statistical significance was determined by one-way ANOVA followed by Tukey’s HSD post-hoc test; different letters indicate significant differences between groups (*p* < 0.05). **B** Total protein extracts, isolated from seedling grown as described, were tested by immunoblotting using anti-HA or anti-phyB antibodies. anti-ACTIN antibody was used as a loading control. Numbers above the blots indicate biological replicates. Numbers below the blots indicate band intensities normalized to the corresponding ACTIN signal

Next, we studied phyD-YFP and phyB-CFP cooperation under pulsed red light irradiation conditions. We administered 1 min of saturating R light pulse in every 3 h for 4 days at 17 °C and compared the photomorphogenic responses. These short irradiation pulses activate phytochromes; however, their signaling strongly depends on the stability of the Pfr conformer during the dark periods between the pulses. We found that under pulsed R irradiation, phyD-YFP alone cannot activate photomorphogenic response whereas phyB-CFP and phyB mediate a partial response relative to the wild-type control. Co-expression of phyD-YFP with phyB-CFP significantly enhances signaling, while co-expression with endogenous phyB restores the response to wild-type levels (Fig. 5A). Next, we measured the accumulation of PHYB-CFP and endogenous PHYB under saturating R pulses (Fig. 5). We have found that R pulses dramatically reduced their protein levels and that co-expression of PHYD-YFP promoted the accumulation of both PHYB-CFP and endogenous PHYB. This protein accumulation pattern was similar to that observed in plants grown under continuous R at low temperatures (Fig. 2).

Thus, we concluded that phyD-YFP and phyB photoreceptors can cooperate at low temperatures under both constant R and short R pulse conditions (Figs. 1 and 5). Furthermore, phyD-YFP promotes phyB accumulation under these conditions, independently of the duration of R irradiation.

## Discussion

To understand the interactions between phyD and phyB, we co-expressed PHYD-YFP with PHYB-CFP transgene and endogenous PHYB in phytochrome-deficient genetic background and examined the efficiency of their light signaling at low and high ambient temperatures, under constant and pulsed R irradiations.

We found that under constant R irradiation phyD-YFP did not inhibit hypocotyl elongation, while phyB-CFP mediated moderately efficient photomorphogenic responses (relative to the aBcde plant) at both temperatures (Fig. 1). At weak R, signaling of phyB-CFP and endogenous phyB was similar, whereas at strong light phyB-CFP mediated weaker photomorphogenesis. Although we cannot exclude that PHYB-CFP is a slightly less efficient photoreceptor due to the CFP-HA tag, our data suggest that signaling efficiency is predominantly defined by the abundance of the photoreceptor. In line with our previous findings (Péter et al. 2024), we observed that phyB protein level was higher in aBcde than in PHYB-CFP plants (Supplementary Fig S3), which explains the moderate PHYB-CFP signaling at strong R. Under this saturating R irradiation at low temperature, the more efficient signaling of the PHYD-YFP + PHYB-CFP plants (relative to the PHYB-CFP plants) can also be explained by the enhanced PHYB-CFP levels (Figs. 2A, 3B, Supplementary Fig S3 and see below).

Consistently with the photomorphogenic phenotypes, under strong R irradiation, similar transcriptome changes occurred in aBcde and PHYB-CFP plants but the transcriptional reprogramming was stronger in aBcde plants at both temperatures (Supplementary Figures S6–S9). The co-expression of phyD-YFP and phyB-CFP enhanced the photomorphogenic response exclusively at low temperatures (Fig. 1). Transcriptome analyses explained this temperature-dependent effect; the R-induced transcriptional responses of PHYB-CFP and PHYD-YFP + PHYB-CFP plants were markedly different only at 17 °C and the transcriptional changes of PHYD-YFP + PHYB-CFP and aBcde plants were much more similar at low than at high temperatures (Fig. 4 and Supplementary Figures S6–S9). Additionally, the PHYD-YFP/aBcde seedlings also exhibited enhanced light responses relative to the aBcde plants only at 17 °C (Supplementary Figure S2). This indicates that the observed co-action does not occur exclusively between PHYD-YFP and PHYB-CFP and that phyD and phyB interact synergistically under prolonged R irradiation in a low temperature-dependent manner.

The molecular basis of this synergism is not clear. Under constant strong R, light signaling mainly depends on the available phytochrome protein pool (Klose et al. 2020). We have recently demonstrated that PHYD-YFP mediates efficient R signaling in the presence of endogenous phyC (PHYD-YFP/abCde plants) at low but not at high temperatures, while neither the PHYD-YFP nor abCde seedlings can mediate photomorphogenic responses on their own (Péter et al. 2024). Here, we show that under R irradiation, PHYD-YFP also synergistically cooperates with phyB in a low temperature-dependent manner. Surprisingly, the molecular basis of PHYD-YFP synergism with phyC is likely different from its synergism with phyB. We have previously shown that at low temperatures under R irradiation, phyC markedly enhances PHYD-YFP protein accumulation; thus, we proposed that this increased PHYD-YFP pool is responsible for the efficient R signaling (Péter et al. 2024). By contrast, phyB does not enhance PHYD-YFP accumulation (Supplementary Fig S5), whereas PHYD-YFP increases PHYB-CFP and phyB protein levels under either continuous R or pulsed R irradiation (Figs. 2, 5 and Supplementary Figures S2 and S3). It will be interesting to test whether phyC also alters phyB protein accumulation (Sánchez-Lamas et al. 2016).

Taken together, our results suggest that PHYD-YFP enhances R signaling by increasing the phyB pool. However, PHYB-CFP levels are comparably enhanced at both low and high temperatures in PHYD-YFP + PHYB-CFP plants (Fig. 2), whereas PHYD-YFP stimulates R signaling only at 17 °C. These findings raise two questions: how does PHYD-YFP enhance PHYB-CFP levels, and why does it promote R signaling only at low temperatures?

Because phyB-CFP can mediate effective photomorphogenesis alone whereas phyD-YFP cannot (Fig. 1 and (Péter et al. 2024), we propose that phyB-CFP is the primary contributor to light signaling in the PHYD-YFP + PHYB-CFP plants. In continuous R light, PHYB-CFP protein level is higher in the presence of PHYD-YFP. It is not due to enhanced transcription, PHYB-CFP mRNA levels are comparable in the single and double transgenic plants (Supplementary Figure S11). However, there is a difference in the stability of these photoreceptors: whereas phyB-CFP is unstable in the light and its abundance decreases under irradiation, phyD-YFP is stable in R (Péter et al. 2024) and Fig. 3). We propose that phyD-YFP stabilizes phyB-CFP, which leads to the accumulation of PHYB-CFP protein and, consequently, to more efficient photomorphogenic responses. Similarly, in PHYD-YFP/aBcde plants, phyD-YFP stabilizes endogenous phyB under R at low temperatures and promotes light signaling, indicating the conserved nature of this functional cooperation (Supplementary Figures S2 and S3).A series of studies demonstrated that phyD and phyB proteins interact directly and form heterodimers (Sharrock and Clack 2004; Clack et al. 2009; Liu and Sharrock 2013). We speculate that phyD-YFP can also form functional heterodimers with both transgenic and endogenous phyB. Indeed, co-immunoprecipitation assays revealed that PHYD-YFP and PHYB form protein complexes at a wide range of temperatures (Supplementary Figure S10). We propose that these heterodimers are more stable than the phyB homodimers in light, thereby facilitating more efficient signaling. Alternatively, phyD-YFP indirectly stabilizes phyB, for example by sequestering the LRB ubiquitin ligases (Ni et al. 2014) that degrades phyB in a light-dependent manner. However, this latter model does not explain how phyD-YFP enhances phyB level without intensifying light signaling at high temperatures.

To explain why phyD-YFP does not stimulate R responses at high temperatures in PHYD-YFP + PHYB-CFP and PHYD-YFP/aBcde plants, we propose that phyD-YFP becomes inefficient at high temperatures (presumably due to improper folding); thus, at 27 °C the heterodimers retain limited functionality. A non-exclusive alternative explanation is that the thermal reversion of PHYD-YFP-phyB heterodimers is much faster than that of the phyB and phyB-CFP homodimers. In this scenario, at high temperatures, the phyB component of the heterodimers reverts rapidly even under continuous R light, meaning that the enhanced PHYB protein levels do not lead to more efficient light responses, highlighting a critical trade-off between total protein abundance and Pfr stability. Enhanced thermal reversion at high temperature can also explain our findings that in the PHYB-CFP plants, PHYB-CFP protein amounts are higher at 27 °C than at 17 °C, whereas the signaling is not obviously more efficient (Figs. 1 and 2). Unfortunately, low signal-to-noise ratios prevented *in planta* thermal reversion rate measurements in these transgenic lines, providing a limitation to the present study.

To address the limitations of direct Pfr/Pr measurements, we used an alternative experimental strategy, in which thermal reversion could play a more relevant regulatory role. We investigated the signaling and the interactions of the phyD-YFP and phyB phytochromes in a R pulse photomorphogenic assay. In this experimental setup, signaling is defined by the rate of degradation due to the short R pulses and the rate of thermal reversion. While degradation regulates the available total phytochrome pool, thermal reversion determines active phytochrome levels by modulating Pfr inactivation in the dark. Although phyD-YFP alone cannot mediate photomorphogenic responses, PHYB-CFP and aBcde plants showed moderate response under R pulses. Notably, phyD-YFP enhanced the R pulse responses of both PHYB-CFP and aBcde plants (Fig. 5A). We expected that short R pulses would only moderately reduce the phytochrome pool, meaning thermal reversion rates would define signaling efficiency. Surprisingly, pulsed R light—much like continuous R—significantly reduced PHYB and PHYB-CFP levels in the absence of PHYD-YFP. Conversely, the presence of PHYD-YFP led to the higher accumulation of both PHYB-CFP and PHYB proteins. Therefore, we propose that under pulsed light, as under continuous light, PHYD-YFP stimulates R signaling by stabilizing PHYB-CFP and PHYB proteins, thereby expanding the active phytochrome pool (Fig. 5B).

## Conclusions

We conclude that phyD-YFP enhances plant light responsiveness through the physical stabilization of the phyB protein at low temperatures. This functional cooperation allows for elevated phyB signaling levels that serve as a basis for enhanced signaling, particularly under low-temperature conditions. Moreover, we previously found that phyD also synergistically interacts with phyC at low temperatures. Collectively, our results reveal that the co-action of phyD with other phytochromes can enhance photomorphogenesis under certain conditions, providing a clear example of how apparently redundant photoreceptors collaborate to ensure developmental plasticity in diverse thermal environments.

## Supplementary Information

Below is the link to the electronic supplementary material.

Supplementary file1 (PDF 1210 KB)

Supplementary file2 (XLSX 245 KB)

Supplementary file3 (XLSX 151 KB)

Supplementary file4 (XLSX 89 KB)

Supplementary file5 (XLSX 131 KB)

Supplementary file6 (XLSX 151 KB)

Supplementary file7 (XLSX 143 KB)

Supplementary file8 (XLSX 44 KB)

Supplementary file9 (XLSX 27 KB)

## Data Availability

All transcriptome data generated and analyzed during the current study are available in the Gene Expression Omnibus repository (https://www.ncbi.nlm.nih.gov/geo/) under the accession number GSE253043.
