# Supplementary material for: Transgenic phytochrome D cooperates with phytochrome B to promote photomorphogenesis at low temperatures
Source: Plant Cell Rep. 2026 Oct 23;45(11):340. doi: 10.1007/s00299-026-04025-y (PMC13694552; doi:10.1007/s00299-026-04025-y)
Supplement: Supplementary file 1 [file 299_2026_4025_MOESM1_ESM.pdf]

Supplementary figures

Transgenic phytochrome D and phytochrome B cooperatively promote photomorphogenesis at low temperatures

Lilla Sípos[1,2], Gábor Grézal[3,4], Csaba Péter[1,2], Dániel Silhavy[1], Ferenc Nagy[1] and András Viczián[1]

[1]Laboratory of Photo and Chronobiology, Institute of Plant Biology, Biological Research Centre, Hungarian Research Network (HUN-REN), Szeged, H-6726 Hungary
[2]University of Szeged, Faculty of Sciences and Informatics, Doctoral School of Biology, Szeged H-6726, Hungary
[3]Synthetic and Systems Biology Unit, Institute of Biochemistry, Biological Research Centre, Hungarian Research Network (HUN-REN), Szeged, H-6726, Hungary
[4]Metabolic Systems Biology Laboratory, HCEMM–BRC, and Genomic Epidemiology Research Group, HUN-REN–BRC–ELTE–SZTE–NNGyK, Szeged, H-6726, Hungary

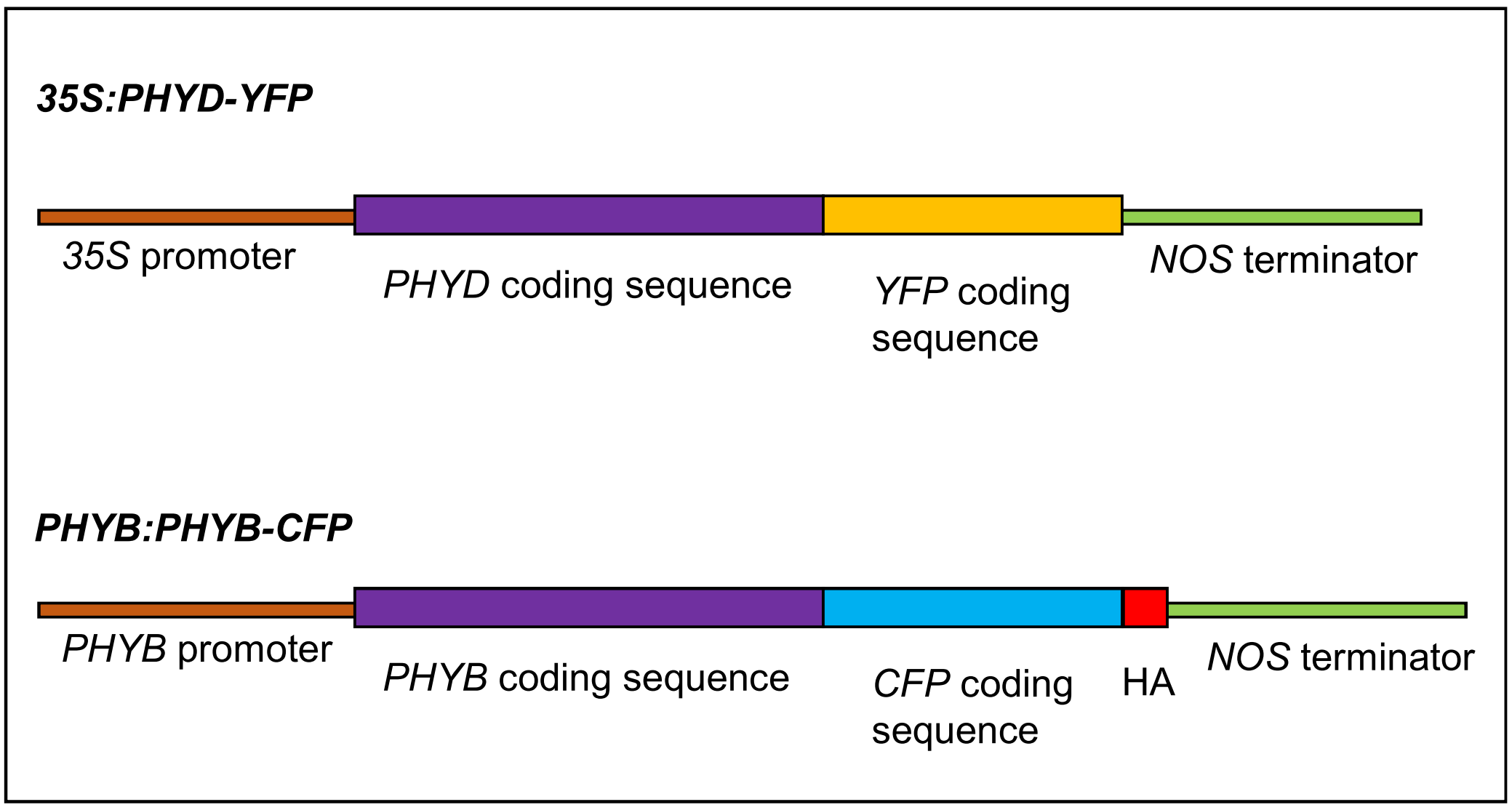

Supplementary Figure S1. Schematic drawing of the genetic constructs used in the study.
The figure is not drawn to scale.

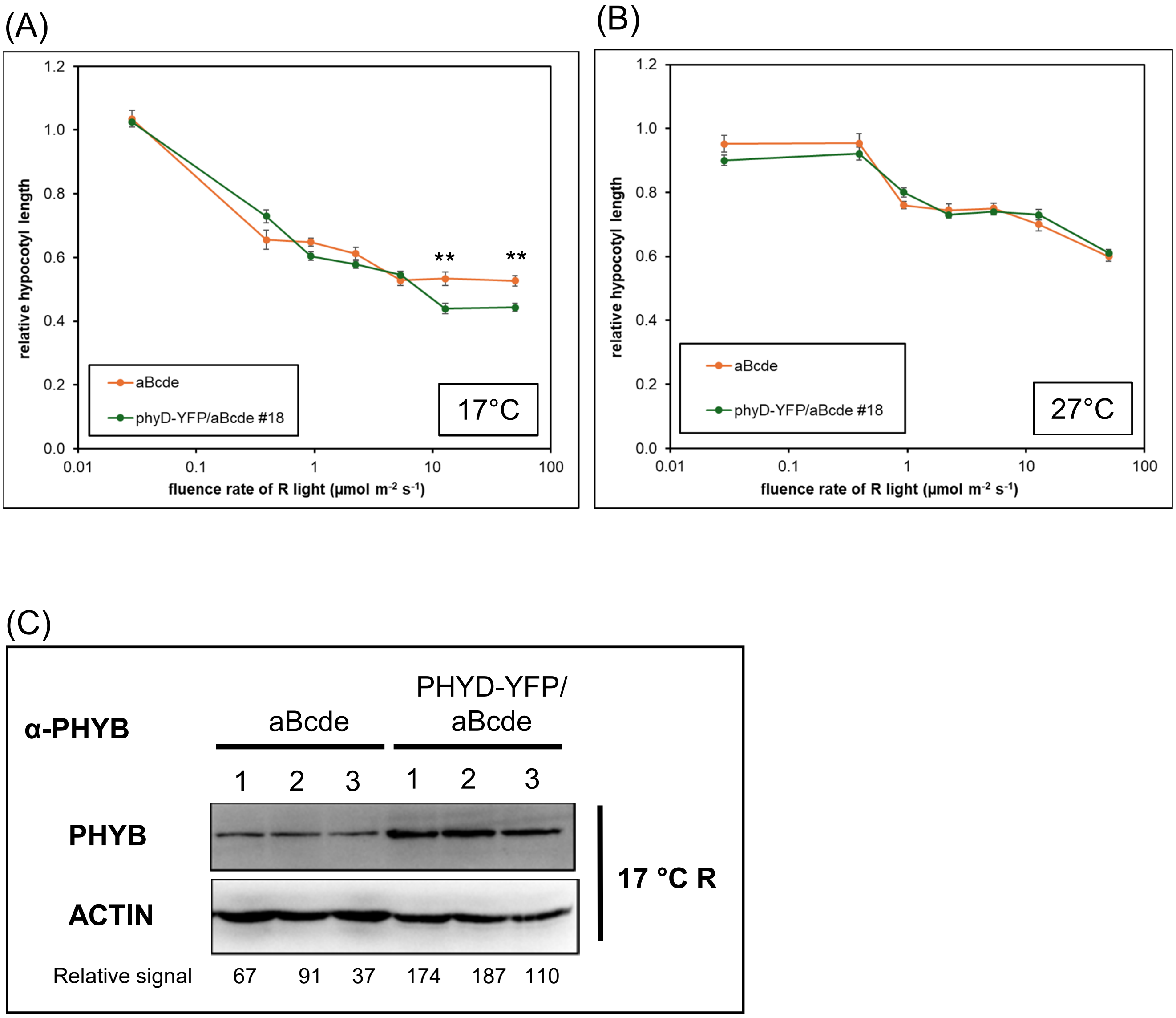

Supplementary Figure S2. PHYD-YFP promotes the photomorphogenesis of phyB and the accumulation of endogenous PHYB protein at low temperatures.
35S:PHYD-YFP/aBcde and aBcde seedlings were grown at for 4 days in darkness or under continuous red light (50 µmol m^{-2} s^{-1}). Hypocotyl lengths of seedlings grown at either 17 °C (A) or at 27 °C (B) were measured and are depicted as relative to the corresponding dark controls. n≥30, error bars indicate standard errors. The marked points show significant differences (Student's t-test, **$p < 0.01$). (C) Total protein extracts were probed via western blot hybridization using anti-PHYB that recognizes endogenous phyB. Hybridization with anti-ACTIN was done as a loading control. Numbers above the blots indicate biological replicates. Numbers below the blots indicate band intensities normalized to the corresponding ACTIN signal.

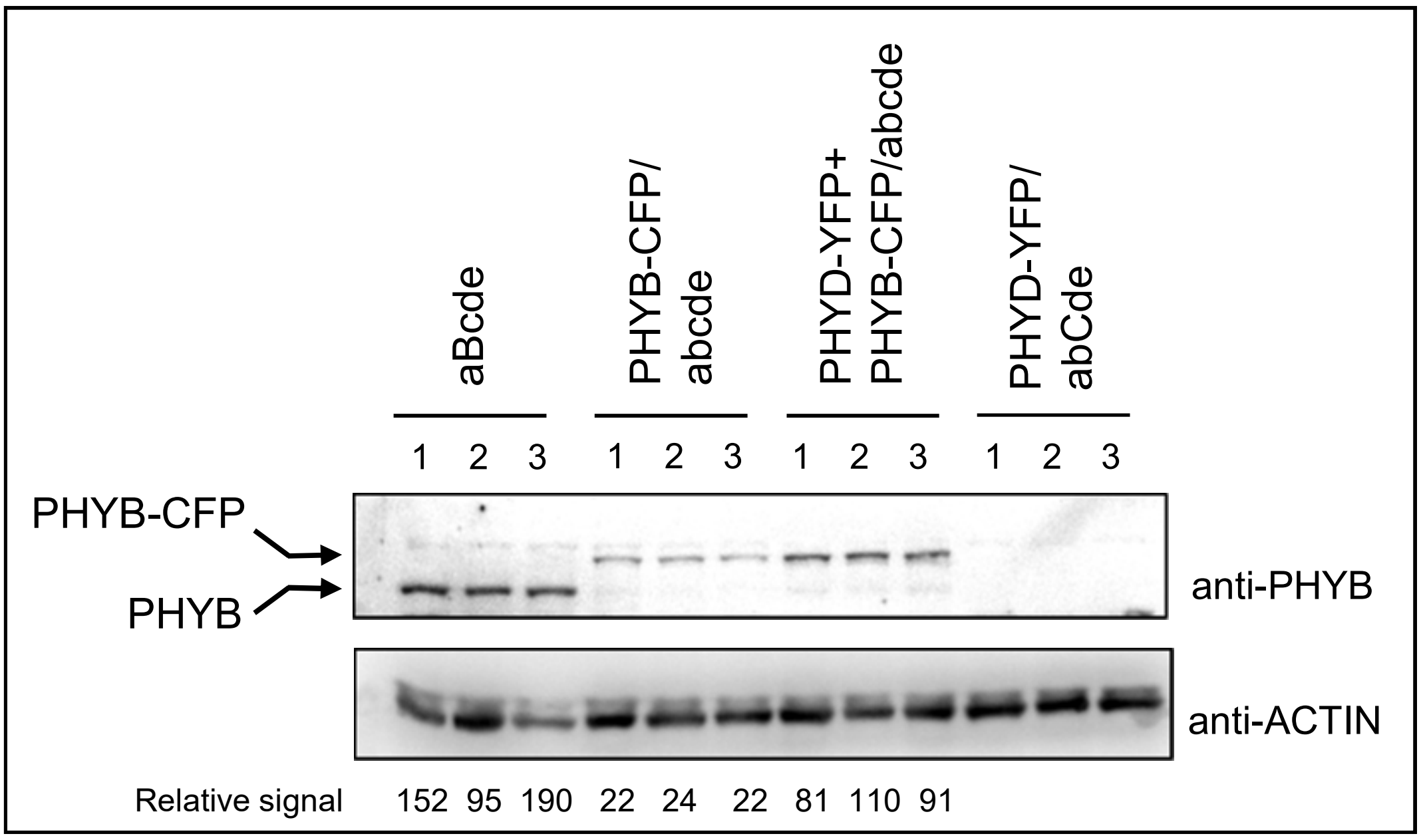

Supplementary Figure S3. Western blot showing the accumulation of PHYB and PHYB-CFP proteins at low temperature.

Seedlings were grown at 17 °C for 4 days in continuous red light (50 µmol m^{-2} s^{-1}). Total protein extracts prepared from aBcde, *PHYB:PHYB-CFP/*abcde, *35S:PHYD-YFP+PHYB:PHYB-CFP*/abcde, and *35S:PHYD-YFP/*abCde were probed with western blot hybridization using an anti-PHYB antibody. The *35S:PHYD-YFP/abCde* sample was included to test the specificity of the anti-PHYB antibody. Hybridization with anti-ACTIN served as a loading control. Numbers above the blots indicate biological replicates. Numbers below the blots indicate band intensity values normalized to the corresponding ACTIN signal.

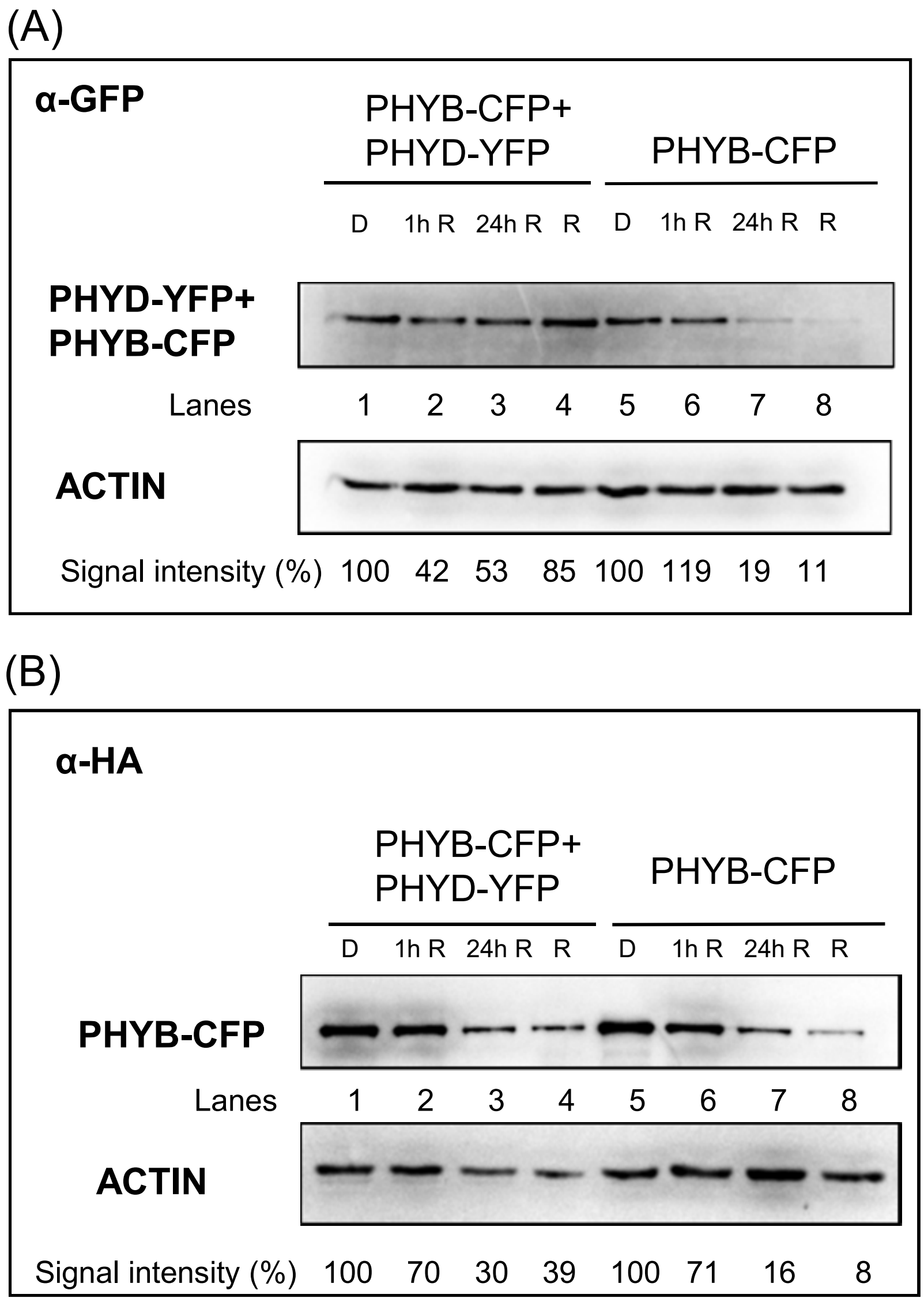

Supplementary Figure S4. High PHYD-YFP stability in red light is responsible for high phytochrome levels in the PHYD-YFP+PHYB-CFP double transgenic plants. Seedlings were grown at 17 °C for 96 h in darkness (D), or for 95 h in darkness followed by 1 h red light (1 h) or 72 h in darkness followed by 24 h of red light irradiation (24 h) or for 96 h in continuous red light (50 µmol m^{-2} s^{-1}). Total protein extracts were analyzed by western blot hybridization using anti-GFP, which detects both PHYD-YFP and PHYB-CFP (A) and using anti-HA antibody, which detects only PHYB-CFP (B). Hybridization with anti-ACTIN was done as a loading control. Numbers below the blots indicate band intensities normalized to the corresponding ACTIN signal, with the dark sample for each genotype set to 100%.

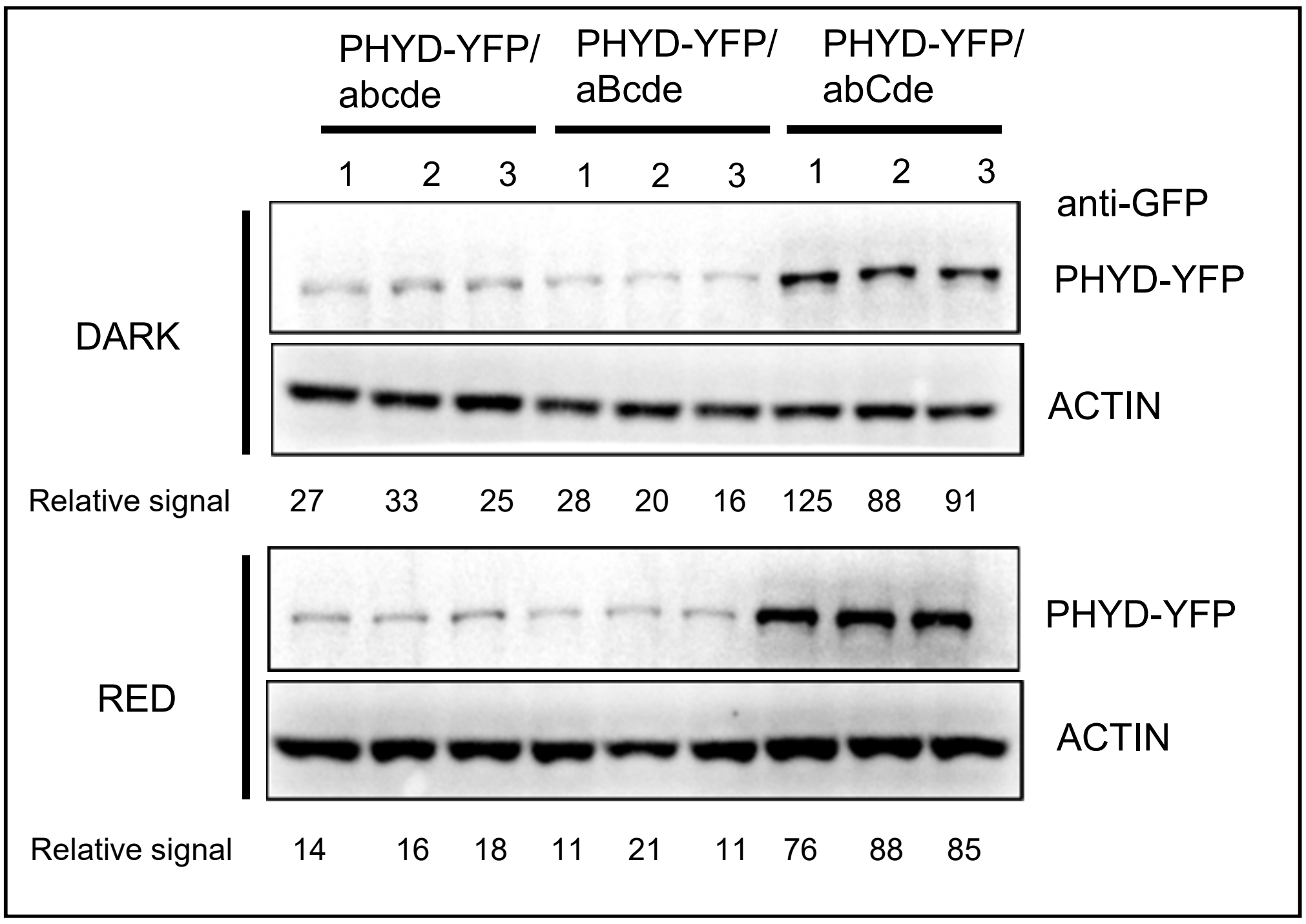

Supplementary Figure S5. PHYD-YFP accumulation is regulated differently by the endogenous *PHYB* and *PHYC*.

Seedlings were grown at 17 °C for 4 days in darkness or under continuous red light (50 μmol m^{-2} s^{-1}). Total protein extracts were probed via western blot hybridization using anti-GFP, which detects PHYD-YFP. Hybridization with anti-ACTIN was done as a loading control. Numbers above the blots indicate biological replicates. Numbers below the blots indicate band intensities normalized to the corresponding ACTIN signal.

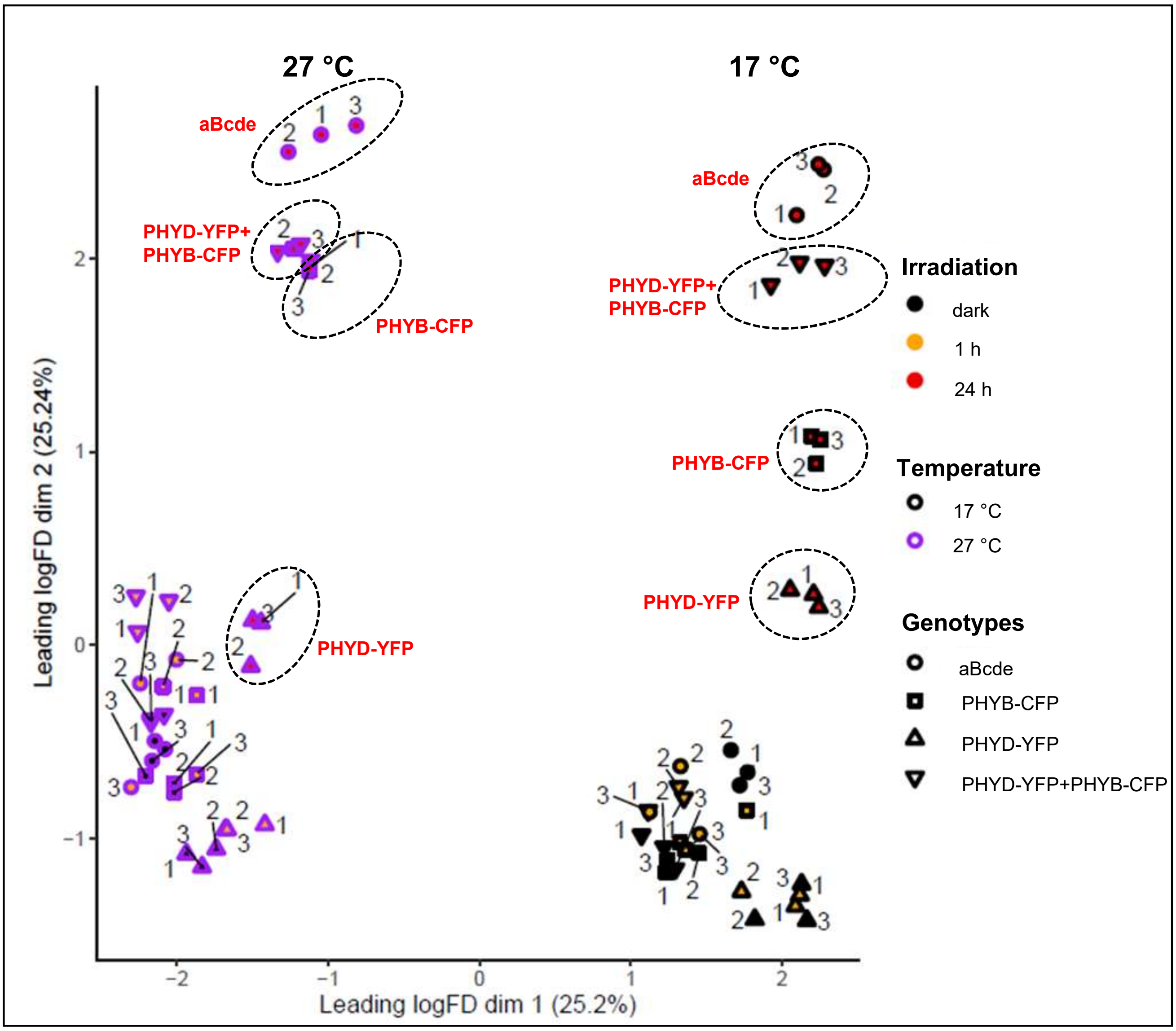

Supplementary Figure S6. Principal Component Analysis (PCA) of transcriptome changes of red light treated plants.

RNA-seq was conducted from samples isolated from 4-day-old aBcde, PHYB-CFP, PHYD-YFP and PHYD-YFP+PHYB-CFP plants grown in the dark at 17 or 27 °C, and then were irradiated with strong red light for 1 or 24 h. For each sample, three repeats were analyzed, these are numbered. The repeats of 24 h irradiated samples are marked with a dotted circles. Note that the repeats are well grouped, the samples from the two temperatures are clearly separated and transcriptome changes are obvious only after 24 h treatment.

A

	17 °C		27 °C	
	**1 h**	**24 h**	**1 h**	**24 h**
**aBcde**	1450	3561	241	2715
**PHYB-CFP**	61	1529	339	2191
**PHYD-YFP+PHYB-CFP**	100	2807	667	2073
**PHYD-YFP**	8	679	10	313

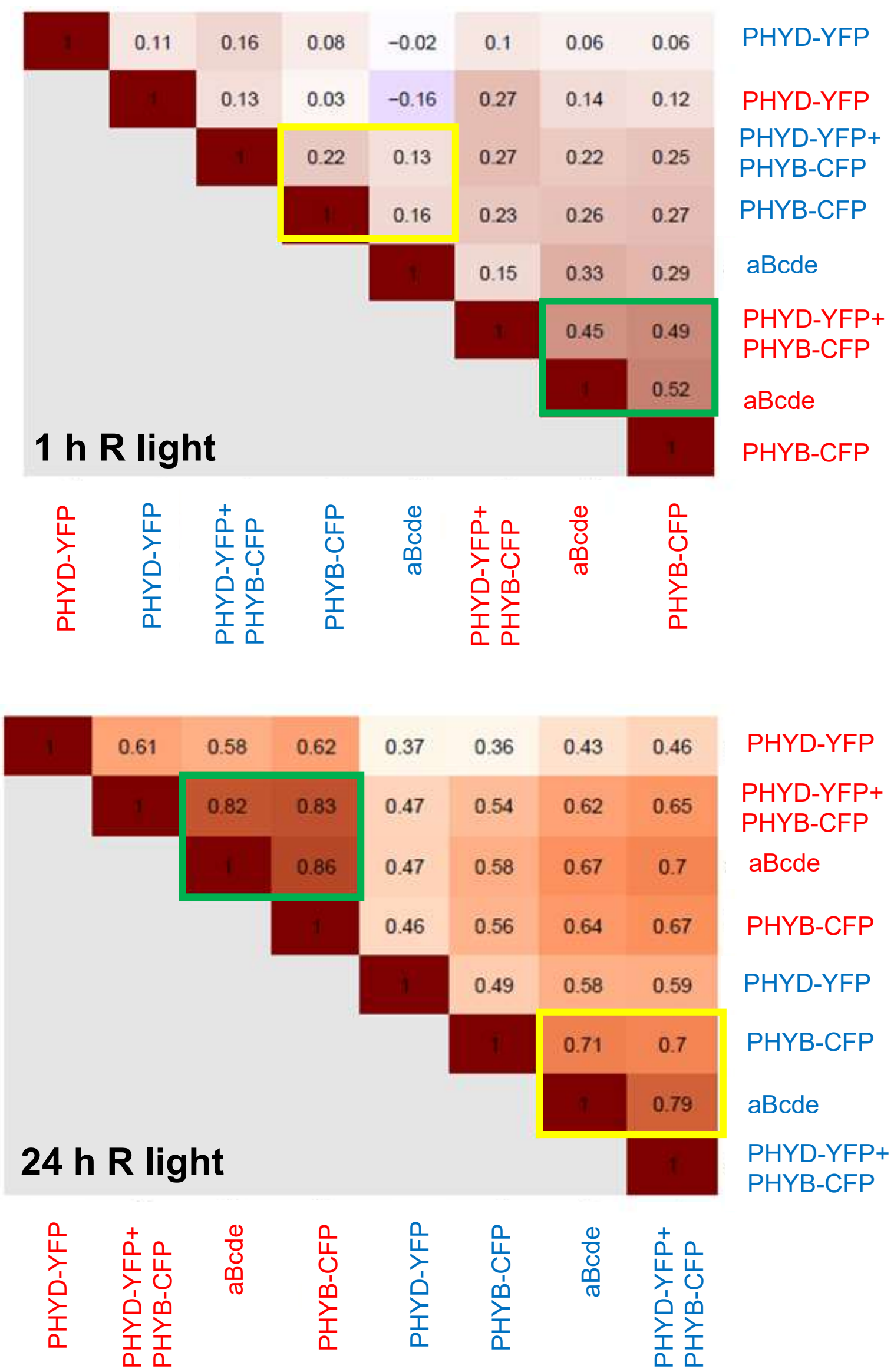

Supplementary Figure S7. Gene expression changes of the red light treated plants. aBcde, PHYB-CFP, PHYD-YFP and PHYD-YFP+PHYB-CFP plants grown in dark at 17 or 27 °C were treated with strong red light (R) for 1 or 24 h. (A) Number of differentially expressed genes (DEGs, genes that show significantly and at least 2-fold expression differences compared to the dark control). (B-C) Pearson correlations of the gene expression changes after 1 h (B) or 24 h (C) R. Correlations of aBcde, PHYB-CFP, and PHYD-YFP+PHYB-CFP plants at 17 and 27 °C are yellow and green framed, respectively. Blue font indicates plants grown at 17 °C, whereas red font depicts plants grown at 27 °C. Note that the correlations are weak after 1 h but strong after 24 h irradiation and that, after 24 h, at low temperature PHYD-YFP+PHYB-CFP correlates better with aBcde than PHYB-CFP.

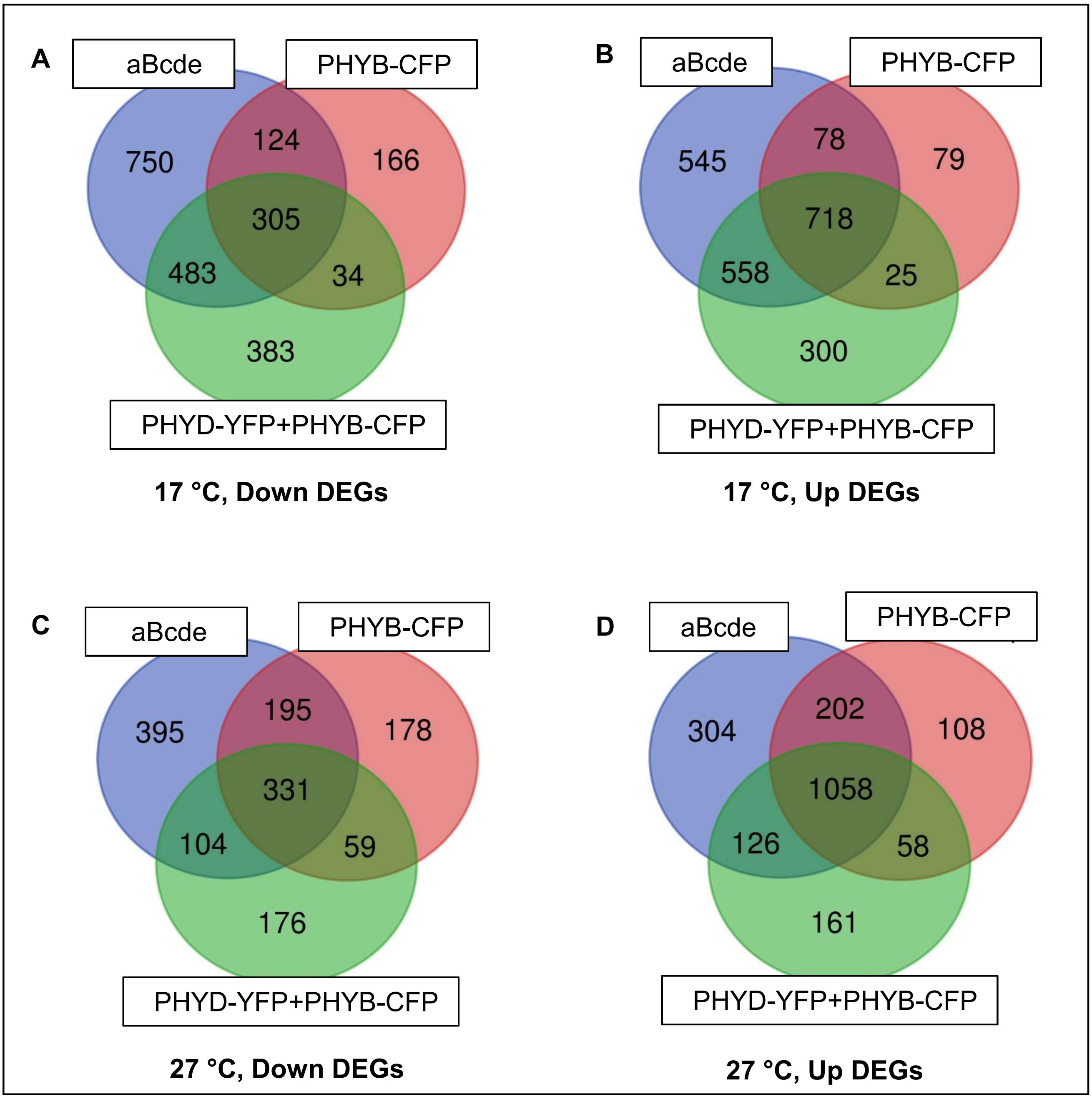

Supplementary Figure S8. VENN-diagram of the differentially expressed genes of the red light treated plants.

The similarity of the DEGs of aBcde, PHYB-CFP and PHYD-YFP+PHYB-CFP plants, which were grown in the dark at 17 or 27 °C, and then were treated with strong red light 24 h. DEGs are differentially expressed genes relative to the dark controls. Comparisons of the downregulated (A and C) and upregulated DEGs (B and D) of plants grown at 17 °C ((A) and (C)) or at 27 °C ((D) and , respectively.

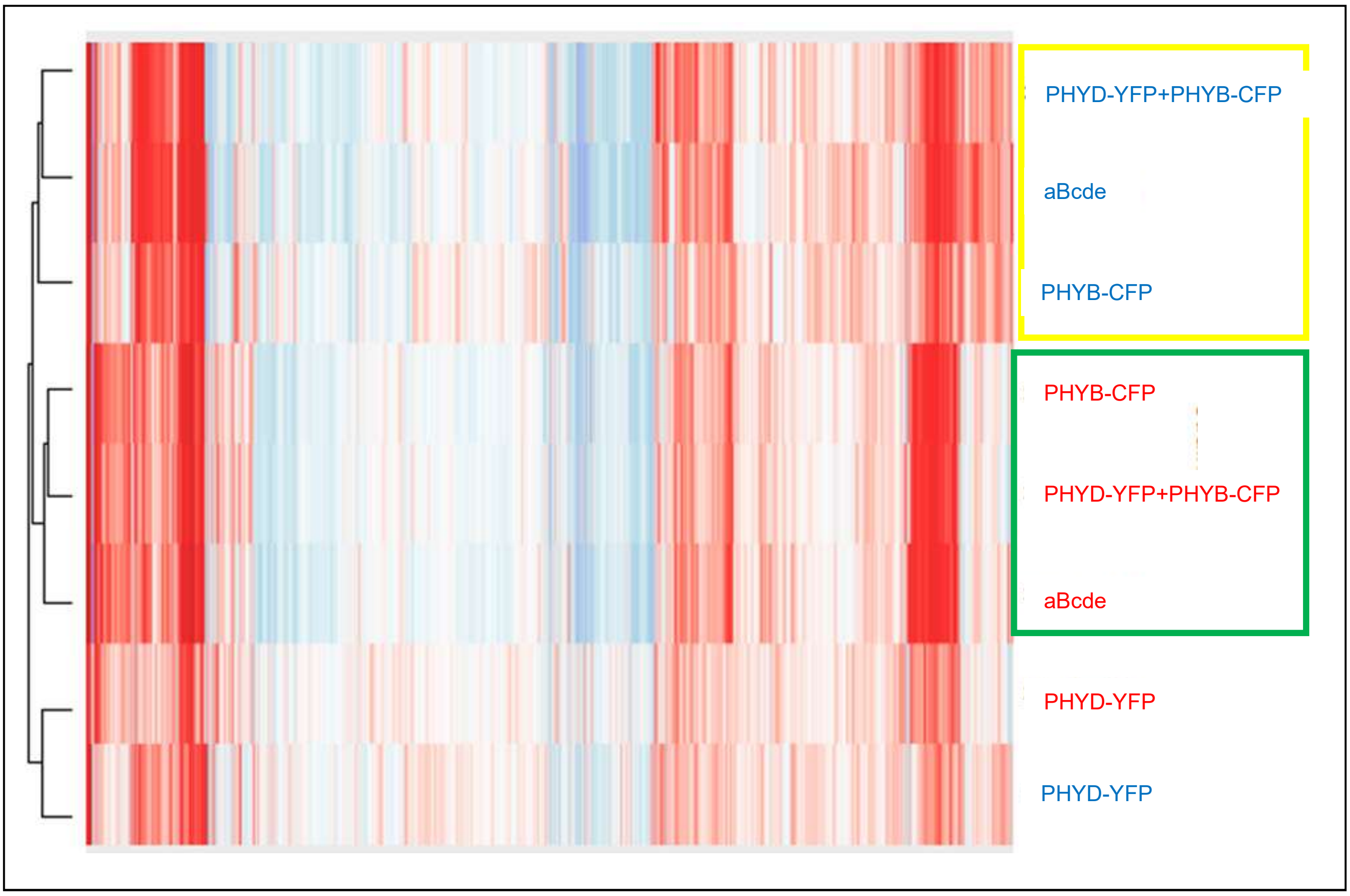

Supplementary Figure S9. Heat-map of gene expression changes in the different red light-treated plants.

The transcriptome changes of 24 h strong red light-treated aBcde, PHYB-CFP, PHYD-YFP and PHYD-YFP+PHYB-CFP plants are compared. Note that the transcriptional changes in the PHYD-YFP+PHYB-CFP plants is most similar to the aBcde plants at 17 °C, and to the PHYB-CFP plants at 27 °C (yellow and green bracketed, respectively). Blue font indicates plants grown at 27 °C, whereas red font depicts plants grown at 27 °C.

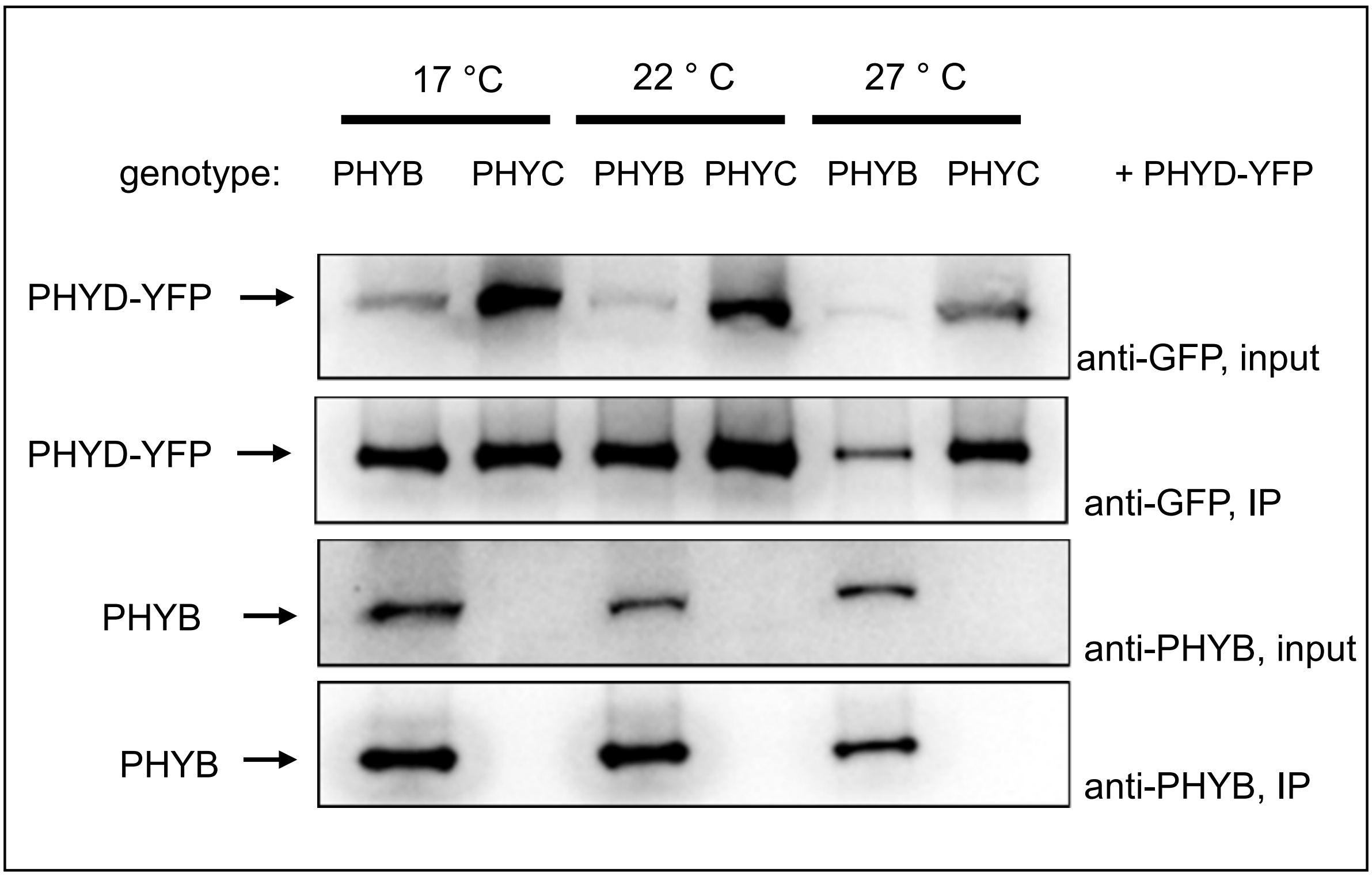

Supplementary Figure S10. Co-immunoprecipitation demonstrates that endogenous PHYB and PHYD-YFP localize to the same protein complexes. Plants were grown for 7 days at 22 °C under a 12-h light / 12-h dark photoperiod, followed by 3 days at the specified temperatures. Total native protein extracts (input) were prepared from *35S:PHYD-YFP*/aBcde and *35S:PHYD-YFP*/abCde plants and subjected to immunoprecipitation (IP) using anti-GFP beads to capture PHYD-YFP-containing protein complexes. Note that PHYB forms complexes with PHYD-YFP at all tested temperatures.

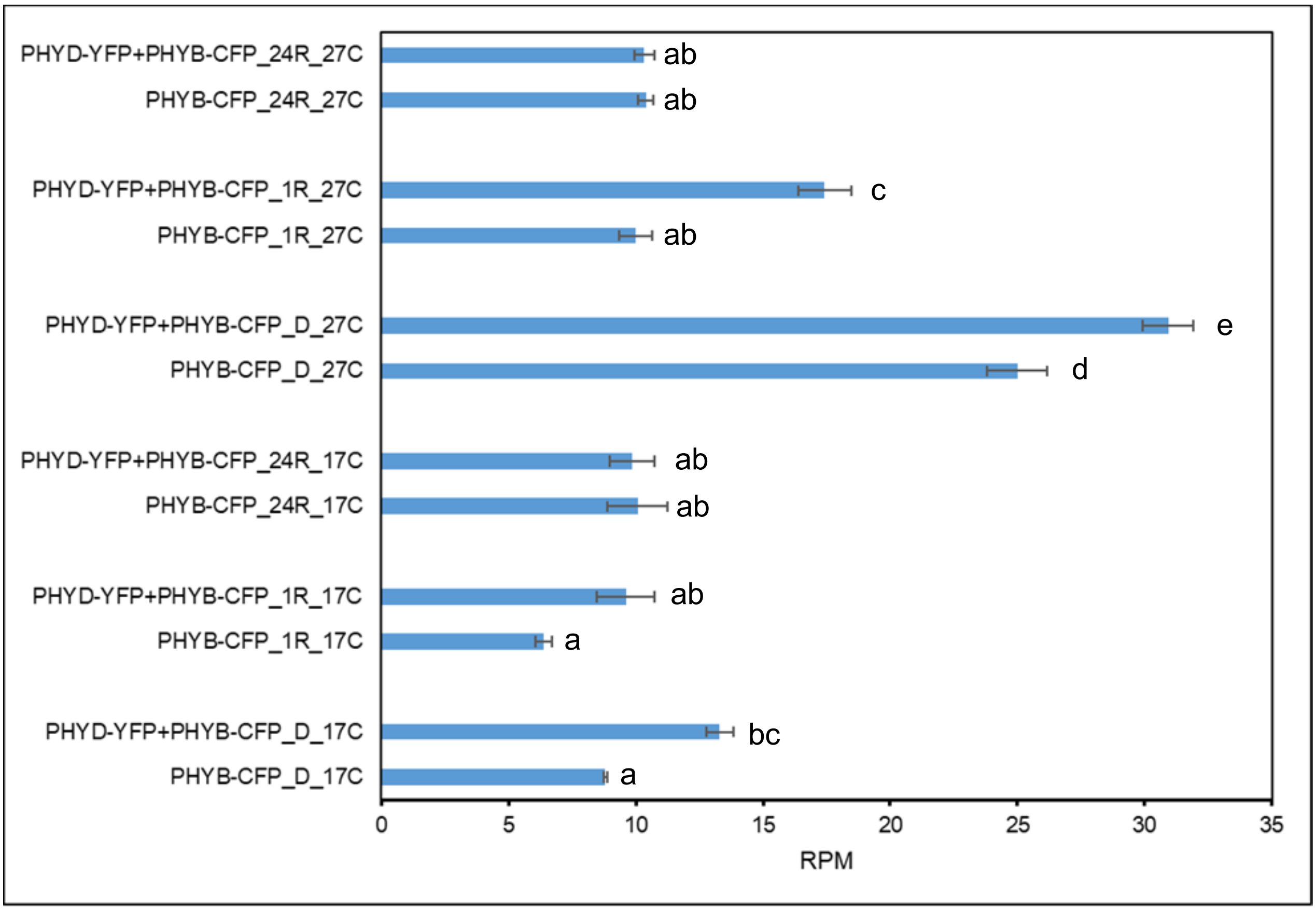

Supplementary Figure S11. ***PHYB-CFP* transgene mRNA expression across genotypes and treatments.**

The transcriptome database (Supplementary Figure S6) was used to estimate *PHYB-CFP* transcript abundance by counting reads containing the sequence 5′-GCGACCATTGTCAACTGCTAGTGGAAGTGGTGACATGATGCTGATGATGCCATAT-3′ and followed by GGGA. To enable comparisons across samples, counts were normalized to the total number of mapped reads in each sample and plotted as reads per million mapped reads (RPM). Data were analyzed using one-way Analysis of Variance (ANOVA) followed by Tukey's Honestly Significant Difference (HSD) post-hoc test (α= 0.05) for multiple comparisons across all groups. Bars represent group means ± standard error (SE) of three biological replicates (N = 3). Groups sharing the same letter are not significantly different from each other ($p > 0.05$), whereas different letters denote statistically significant differences ($p < 0.05$). Note that *PHYB-CFP* mRNA accumulated to slightly but significantly higher levels in the double than in the single transgenic plants in the dark; however, after 24 h of strong R light, no difference can be observed, indicating that its expression does not differ under continuous light.
